# Taxonomy of *Strongyloides* in humans, dogs and cats: a comprehensive review from morphology to molecular and population genetics

**DOI:** 10.1017/S003118202610167X

**Published:** 2026-04

**Authors:** Huan Zhao, Constantin Constantinoiu, Richard Stewart Bradbury

**Affiliations:** 1 School of Public Health and Tropical Medicine, College of Medicine and Dentistry, James Cook University, Townsville, QLD, Australia; 2 School of Veterinary Science, College of Science and Engineering, James Cook University, Townsville, QLD, Australia

**Keywords:** cats, dogs, humans, molecular phylogenetics, morphology, pets, population genetics, *Strongyloides*, strongyloidiasis, taxonomy

## Abstract

The genus *Strongyloides* (Nematoda; Strongyloididae) comprises over 50 species of nematodes parasitic in terrestrial vertebrates, including humans (*Homo sapiens*), dogs (*Canis lupus familiaris*) and cats (*Felis catus*). Taxonomy of the genus has been shaped by over a century of morphological research, with the most widely adopted framework established in the late 1980s. Advances in molecular genetics have increasingly revealed cryptic diversity and yielded new insights into interspecific and intraspecific relationships within the genus. Despite the rapid expansion of molecular genetic data over the past decade, particularly for *Strongyloides* spp. infecting humans and companion animals, a synthesis of these findings remains lacking. Here, we review historical and contemporary literature on the taxonomy of *Strongyloides* spp. infecting humans (*Strongyloides stercoralis, Strongyloides fuelleborni*), dogs (*S. stercoralis*, including host-specific lineages and cryptic taxa) and cats (*Strongyloides felis, Strongyloides planiceps, Strongyloides tumefaciens* and *S. stercoralis*). We provide an updated overview of taxonomic histories, host ranges and key morphological features for genus identification and species differentiation, along with a synthesis of available molecular taxonomic data informed by phylogenetic and population genetic studies. This work is intended to serve as a renewed reference for researchers, diagnosticians and clinicians working with *Strongyloides* spp. in medical and veterinary contexts, supporting accurate diagnosis and guiding future taxonomic research on these nematodes.

## Graphical abstract

**Figure:**
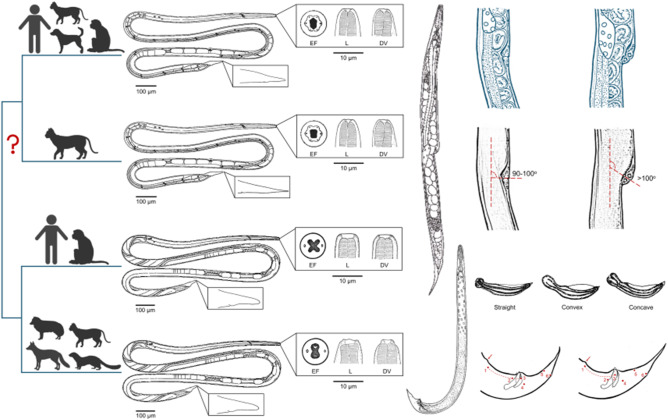

## Introduction

The genus *Strongyloides* (Nematoda; Strongyloididae) comprises over 50 species of parasitic nematodes infecting a wide range of vertebrates, including humans (*Homo sapiens*) and their major companion animals, dogs (*Canis lupus familiaris*) and cats (*Felis catus*) (Speare, 1986; Al-Jawabreh et al., 2024a). Despite their medical and veterinary significance, few sources provide comprehensive taxonomic analyses of *Strongyloides* spp. in these hosts, and those that do are now several decades old (Little, 1966a, b; Speare, 1986, 1989; Grove, 1996). Moreover, no single reference has systematically collated the molecular genetic data accumulated over the past 4 decades. This review aims to fill that gap by providing an updated synthesis of morphological and molecular evidence relevant to *Strongyloides* infecting humans, dogs and cats, to support researchers and diagnosticians working with these parasites in both medical and veterinary contexts.

Two species are known to infect humans: *Strongyloides stercoralis* (Bavay, 1876) and *Strongyloides fuelleborni* (von Linstow O, 1905). The latter is currently divided into 2 subspecies, *Strongyloides fuelleborni* subsp. *fuelleborni* (von Linstow O, 1905) and *Strongyloides fuelleborni* subsp. *kellyi* (Viney et al., 1991). *S. stercoralis* has a cosmopolitan distribution but occurs predominantly in the tropics and sub-tropics, with an estimated 613.9 million people (95% CI: 313.1–910.1) infected globally as of 2017 (Buonfrate et al., 2020). *S. f. fuelleborni* parasitizes Old World non-human primates (NHPs) (Zhao et al., 2025a, b). It has been reported in humans from sub-Saharan Africa, Southeast Asia and South Asia (Pampiglione and Ricciardi, 1972b; Hira and Patel, 1977, 1980; Hasegawa et al., 2010; Thanchomnang et al., 2017; Barratt et al., 2019; Janwan et al., 2020; de Ree et al., 2024; Zhao et al., 2025d), and most recently, Papua New Guinea (PNG) (Zhao et al., 2025c). *S. f. kellyi* appears restricted to New Guinea, where it has been associated with fatal protein-losing enteropathy in infants (Muller et al., 1987; Ashford et al., 1992; Bradbury, 2021). However, recent molecular analyses have challenged current assumptions regarding its taxonomy and distribution (Zhao et al., 2025c). Two other species, *Strongyloides procyonis* (Little, 1966b) and *Strongyloides ransomi* (Kotlan and Vajda, 1934), have been shown to establish transient infections in humans under experimental conditions only and are therefore not considered further in this review.

In dogs, *S. stercoralis* is the only species known to establish natural infections. Its taxonomic relationship to the human-infecting *S. stercoralis* remains debated. Early experimental evidence suggested that human- and dog-derived *S. stercoralis* may represent distinct species, with the canine form historically referred to as ‘*Strongyloides canis*’ (Brumpt, 1922; Augustine, 1940). This hypothesis has gained renewed interest following the identification of a dog-specific *S. stercoralis* genotype (*cox1* lineage B) (Jaleta et al., 2017; Nagayasu et al., 2017; Barratt et al., 2019). Genomic analyses now indicate that dog and human *S. stercoralis* are genetically divergent but not completely reproductively isolated, with evidence of occasional introgression (de Ree et al., 2024; Liu et al., 2025). Additionally, a cryptic *Strongyloides* genospecies has been reported in dogs from northern Australia (Beknazarova et al., 2019). Although *S. f. fuelleborni* (Sandground, 1925b) and *Strongyloides procyonis* (Little, 1966b) can infect dogs experimentally, these species have not been detected in natural canine infections.

Four species of *Strongyloides* have been reported in cats: *Strongyloides felis* (Chandler AC, 1925), *Strongyloides planiceps* (syn. *Strongyloides cati*) (Rogers, 1943), *Strongyloides tumefaciens* (Price and Dikmans, 1941) and *S. stercoralis* (Brown, 1969). These species remain poorly studied. *S. felis* has been described in only a handful of studies, most published over 40 years ago (Chandler AC, 1925; Speare and Tinsley, 1986, 1987; Jitsamai, 2019). Identification of this species is challenging due its close morphological similarity to *S. stercoralis* (Speare, 1986) and the lack of sequencing data for phylogenetic analysis (Zhao et al., 2025a). *S. planiceps* appears to infect primarily wild felids and canids, with sporadic cases in domestic cats (Rogers, 1939; Horie et al., 1981; Fukase et al., 1983, 1985; Sato et al., 2006; El-Seify et al., 2017). *S. tumefaciens* is distinguished by producing characteristic colonic nodules in cats (Price and Dikmans, 1941). However, most feline surveys reporting *S. stercoralis* or *S. tumefaciens* have lacked definitive species confirmation (Zhao and Bradbury, 2024). The only molecularly confirmed case of *S. stercoralis* infection in cats exhibited colonic pathology atypical for this species but consistent with *S. tumefaciens* (Wulcan et al., 2019), raising questions as to whether these taxa are synonymous or represent distinct species with overlapping pathology.

Morphology remains the principal taxonomic tool for defining species of *Strongyloides*. The morphological criteria established by early researchers, particularly Little (1966a) and Speare (1986), have traditionally served as the gold standard for *Strongyloides* identification and diagnosis in medical and veterinary laboratories. Unfortunately, much of this foundational literature is now out of print and accessible only through inherited physical copies held by a small number of researchers. Although the rise of molecular diagnostics has shifted focus away from morphology-based identification (Bradbury et al., 2022), morphology remains the cornerstone of parasite taxonomy and continues to be the most practical diagnostic tool in resource-limited settings, where the burden of infection is often highest (Buonfrate et al., 2020). Efforts must be made to preserve and pass on morphological knowledge and expertise to future generations of parasitologists and diagnosticians.

Molecular genetics play an increasingly important role in nematode taxonomy (Thaenkham et al., 2022). Phylogenetic and population genetic analyses allow the identification of cryptic diversity, clarify host specificity and resolve taxonomic ambiguities that morphology alone cannot address (González, 2025). The integration of morphological and molecular datasets has recently enabled major taxonomic revisions in other helminth genera, including the reassignment of *Mansonella perstans* and *Mansonella* sp. ‘DEUX’ (Rodi et al., 2023), and the separation of *Dirofilaria asiatica* from *D. repens* (Colella et al., 2025). In clinical diagnostics, DNA barcoding facilitates rapid and accurate detection and can be applied to complex samples unsuitable for traditional morphological analysis (González, 2025).

Over recent decades, *Strongyloides* taxonomy has been increasingly informed by molecular genetic data. Two reviews have summarized advances in molecular genotyping (Bradbury et al., 2021) and omics-based approaches (Al-Jawabreh et al., 2024b); however, both focused specifically on human-infecting species. Given the rapid expansion of sequence databases encompassing both human- and animal-infecting *Strongyloides*, a comprehensive synthesis across these host groups is now warranted to guide future taxonomic studies in medical and veterinary contexts.

Herein, we review decades of progress in the taxonomy of *Strongyloides*, with a focus on species infecting humans, dogs and cats. This focus reflects their primary medical and veterinary importance, their potential roles in zoonotic transmission and the recent expansion of molecular genetic data available for these hosts. Our aim is to provide a comprehensive and updated reference for parasitologists and diagnosticians by consolidating dispersed morphological and molecular evidence to support accurate species identification and inform future taxonomic studies. First, we provide an overview of these species, including their taxonomic history and host range. Second, we review morphological criteria applied to *Strongyloides* taxonomy and present detailed descriptions and comparative diagnostic features for genus identification and species differentiation. Third, we synthesize existing phylogenetic and population genetic evidence pertinent to the molecular taxonomy of *Strongyloides*.

## Overview of *Strongyloides* species infecting humans, dogs and cats

### Strongyloides stercoralis

#### Taxonomic history

In 1876, Louis Normand, a physician at the Naval Hospital in France, identified a small worm (∼0.25 mm in length) in the faeces of soldiers returning from Cochin-China (present-day Vietnam) with severe diarrhoea (Bavay, 1876). Bavay (1876), his colleague, named it *Anguillula stercoralis*. During a subsequent autopsy of a soldier with similar symptoms, Normand discovered a larger worm (∼2 mm) in the small intestine, which Bavay (1877) identified as a separate species, *Anguillula intestinalis*. Shortly thereafter, Bavay (1877) observed larvae in cultured stools, mistakenly attributing them to *A. intestinalis*. These forms were later recognized as the rhabditiform larvae (from Greek *rhabdos*, ‘rod’, referring to the rod-like, 3-part oesophagus characteristic of the feeding larval stages), parasitic adults and filariform larvae (from Latin *filum*, ‘thread’, and *forma*, ‘shape’, referring to the elongated/cylindrical oesophagus) of a single species, now known as *Strongyloides stercoralis* (Grassi, 1879). The relationship between these stages was clarified by Grassi and Parona in 1878, who established the genus *Strongyloides* and named the parasite *Strongyloides intestinalis* (Grassi, 1879). In 1881, Perroncito cultivated free-living adults from larvae and referred to them as *Pseudorhabditis stercoralis*, a designation later corrected by Leuckart, who confirmed they belonged to the same species (Speare, 1986). The nomenclature was ultimately resolved by Stiles and Hassall in 1902, who assigned the definitive name *Strongyloides stercoralis* (Stiles and Hassall, 1902).

#### Host range

Since its original description in humans (Bavay, 1876), *S. stercoralis* has also been identified in dogs (Fulleborn, 1914), cats (Brown, 1969) and NHPs (Penner, 1981). The taxonomic status of dog-infecting *S. stercoralis* remains unresolved. Although human and canine strains of *S. stercoralis* are morphologically indistinguishable, early cross-infection experiments showed that human-derived strains did not consistently establish long-term infections in dogs and vice versa (Fulleborn, 1914; Fülleborn, 1927; Sandground, 1928; Augustine and Davey, 1939; Galliard, 1939, 1951; Sandosham, 1952; Grove and Northern, 1982). More recently, population genetic/genomic studies have supported the existence of a dog-specific lineage (*cox*1 lineage B), along with a separate lineage shared among humans, dogs, cats and NHPs (*cox*1 lineage A) (Jaleta et al., 2017; Nagayasu et al., 2017; Barratt et al., 2019; Bradbury et al., 2021). It has been proposed that human-infecting *S. stercoralis* likely originated in wild canids and adapted to humans following the domestication of dogs (Nagayasu et al., 2017; Liu et al., 2025). Evidence of occasional introgression between the human and canine lineages suggests they are not fully reproductively isolated (de Ree et al., 2024; Liu et al., 2025).

The occurrence of *S. stercoralis* in cats remains somewhat enigmatic. Historically, *S. stercoralis* was not considered a natural parasite of felines, as early experimental infections of cats using human- or dog-derived isolates either failed to establish or resulted in only transient patency (Tuira, 1919; Sandground, 1925b; 1926a,b, 1928; Augustine and Davey, 1939; Kadhim, 1968). Although numerous reports have described *S. stercoralis* infection in cats, few included species-level confirmation (Zhao and Bradbury, 2024). The first unequivocal identification was reported by Wulcan et al. (2019), in which phylogenetic analysis of a 522 bp *cox*1 fragment placed the isolate within the human-dog-shared lineage of *S. stercoralis*.

In 1935, Mirza and Narayan isolated an *S. stercoralis*-like parasite from the intestine of an Arctic fox (*Vulpex alopex*) (Mirza and Narayan, 1935). Morphometric analysis showed that the parasite had a shorter tail (∼40 µm) but was otherwise indistinguishable from *S. stercoralis* in humans. Based on this, they proposed it as a variety of *S. stercoralis*, naming it *S. stercoralis* var. *vulpi* (Mirza and Narayan, 1935). This parasite has not been reported in foxes since, and it remains unclear whether foxes are true natural hosts. A separate species, *Strongyloides vulpis*, was described from red foxes (*Vulpes vulpes*) by Petrov in 1940 (Speare, 1986), but original descriptions are now inaccessible. As a result, the taxonomic validity of both *S. stercoralis* var. *vulpi* and *S. vulpis* remains unresolved.

### Strongyloides fuelleborni fuelleborni

#### Taxonomic history

In 1905, von Linstow O (1905) proposed the name *Strongyloides fuelleborni* for a species identified in chimpanzees (*Pan troglodytes*) and yellow baboons (*Papio cynocephalus*) in Africa. In his original description, he reported that this species characteristically shed larvae rather than eggs in faeces; however, it was later clarified that he had intended the opposite. Chandler AC (1925) regarded *S. fuelleborni* as a variant of *Strongyloides papillosus* (Wedl, 1856) Ransom, 1911, whereas Sandground (1925b) and Goodey (1926) considered it a distinct species based on the morphology of the free-living female, particularly the prominent vulvar lips and the narrowing of the body immediately posterior to the vulva.

In 1923, Hung and Höppli (1923) described *Strongyloides simiae* from Asian macaques (*Macaca* sp.), distinguishing it from *S. fuelleborni* and *S. cebus* by the presence of cuticular striations in parasitic females. However, subsequent studies revealed that transverse cuticular striations are common across all *Strongyloides* species, rendering these morphological differences insufficient to support *S. simiae* as a separate species. Consequently, *S. simiae* is regarded as a junior synonym of *S. fuelleborni* (Speare, 1986).

The discovery and subsequent investigations of a *S. fuelleborni*-like species in PNG led to a taxonomic revision of *S. fuelleborni*. The New Guinea parasite was designated *S. fuelleborni kellyi* in 1991, while the original African species described by von Linstow has since became *S. fuelleborni fuelleborni* (Viney et al., 1991). However, this subspecific distinction has recently been challenged by molecular data showing that *S. f. kellyi* shares genotypes with the Asian clade of *S. f. fuelleborni*, suggesting they are taxonomically identical (Zhao et al., 2025c). While these findings point to a synonymy between *S. f. kellyi* and Asian *S. f. fuelleborni*, further morphological and genomic comparisons of isolates from Asia, Africa and the Pacific are needed before any formal taxonomic conclusions can be drawn (Zhao et al., 2025c).

#### Host range

Humans and NHPs are the only confirmed natural hosts of *S. f. fuelleborni* (Speare, 1986). Experimental infections with *S. f. fuelleborni* have been attempted in dogs (*Canis lupus familiaris*), cats (*Felis catus*), brown rats (*Rattus norvegicus*), house mice (*Mus musculus*) and guinea pigs (*Cavia porcellus*), but only dogs developed patent infections, which were short-lived and self-limiting (Sandground, 1925b; Rego, 1972).

### Strongyloides fuelleborni kellyi

#### Taxonomic history

During a 1971 parasitological survey in Kiunga, PNG, Allen Kelly detected *Strongyloides* eggs in human stool samples (Kelly and Voge, 1973). In some children, this infection was associated with a severe protein-losing enteropathy known as swollen bell syndrome (SBS). This condition was characterized by the presence of large numbers of *Strongyloides* eggs in stool, often in long mucous strings. In other children, eggs were passed individually, and there were no apparent signs of disease, despite high worm burdens. As it was assumed that only a single egg-producing *Strongyloides* sp. infected humans in PNG, an unknown co-factor was proposed to explain the development of SBS in some infants with heavy infections but not in others (Ashford et al., 1992).

Subsequent morphological analysis of adult *Strongyloides* from PNG children revealed a close resemblance to *S. fuelleborni* von Linstow, 1905. However, the absence of NHPs on the island of New Guinea, combined with the paucity of reported *S. fuelleborni* infections in humans from regions between Africa and New Guinea at the time, left the identity of this *S. fuelleborni*-like nematode (*S. cf. fuelleborni*) unresolved (Viney et al., 1991; Ashford et al., 1992).

Using scanning electron microscopy, Viney et al. (1991) observed subtle morphological differences between *S. fuelleborni* isolated from African NHPs and *S. cf. fuelleborni* from PNG humans, specifically in the peri-vulval cuticle of parasitic females and the position of the phasmidial pore in free-living males. A parallel isoenzyme electrophoretic analysis showed that most *S. cf. fuelleborni* isolates (22/26) grouped with African *S. fuelleborni*; however, 4 isolates unexpectedly clustered with *S. ransomi* from local pigs (Viney and Ashford, 1990). The authors speculated that this may have resulted from participants submitting pig faeces in place of their own (Viney and Ashford, 1990). Notably, the Asian clade of *S. fuelleborni* was not included in these analyses (Viney and Ashford, 1990; Viney et al., 1991). Nevertheless, based on the available data, Viney et al. (1991) designated *S. cf. fuelleborni* as a subspecies of *S. fuelleborni*, naming it *S. f. kellyi* in honour of its discoverer, Allen Kelly.

This taxonomic designation was later challenged by Dorris et al. (2002), who conducted phylogenetic analysis of a 330 bp region of *18S rRNA* and found that *S. f. kellyi* clustered more closely with *S. venezuelensis* and *S. ransomi* than with *S. f. fuelleborni*, prompting calls to elevate it to species status. However, these findings have been criticized due to the use of formalin-fixed specimens, which are prone to DNA degradation and sequencing artefacts, particularly given the limitations of early molecular techniques (Zhao et al., 2025c). A subsequent genotyping study based on the *18S rRNA* hypervariable regions HVR-I (432 bp) and HVR-IV (252-259 bp) similarly identified 1 PNG human isolate (1/8) clustering with *S. venezuelensis* and *S. ransomi*, distinct from *S. f. fuelleborni* (Zhao et al., 2025c). However, the majority of PNG isolates (7/8) grouped within the Asian clade of *S. f. fuelleborni* (Zhao et al., 2025c). The authors postulated the existence of 2 genetically distinct *Strongyloides* nematodes in PNG: *S. f. kellyi* which is likely synonymous with the Asian-Pacific clade of *S. f. fuelleborni*, and a second, undescribed species corresponding to the genospecies identified by Dorris et al. (2002), closely related to *S. ransomi* of pigs. Notably, *S. ransomi* causes a protein-losing enteropathy in piglets similar to SBS (Zhao et al., 2025c), and this second egg-producing *Strongyloides* sp. infecting infants in PNG may explain the previously confusing epidemiology of SBS in that nation.

#### Host range

Humans are the only known hosts of *S. f. kellyi* in New Guinea. Prior to the molecular era, investigations into potential zoonotic reservoirs, including pigs, chickens and dogs, found no evidence of infection in these animals (Viney et al., 1991). Given recent taxonomic insights suggesting that *S. f. kellyi* is likely synonymous with the Asian-Pacific clade of *S. f. fuelleborni* (Zhao et al., 2025c), such findings are not unexpected. Ashford et al. (1992) proposed that *S. fuelleborni* has adapted to exclusive human-to-human transmission in New Guinea without an NHP reservoir, a hypothesis supported by experimental and epidemiological findings from Africa (Pampiglione and Ricciardi, 1972a; Hira and Patel, 1980). However, whether humans are the only natural reservoir in New Guinea remains to be confirmed. This question is particularly relevant given reports of invasive NHP species in parts of the Pacific, including New Guinea (Kemp and Carter, 2022), which could act as mobile zoonotic reservoirs if they come into contact with humans.

The novel, undescribed genospecies identified by Zhao et al., (2025c) and Dorris et al. (2002) may have a zoonotic reservoir in local animals, particularly pigs, given its close genetic relationship to the porcine parasite *S. ransomi* and the relative ubiquity of domestic pigs in PNG. Future investigations into this nematode in both humans and potential animal reservoirs in New Guinea should employ molecular or advanced morphological tools capable of differentiating *Strongyloides* spp.

### Strongyloides felis

#### Taxonomic history

*Strongyloides felis* was first described by Chandler AC (1925) in cats from Kolkata, India. Chandler AC (1925) suspected that *S. felis* might be a variety or subspecies of *S. stercoralis*, tentatively naming it *S. stercoralis* var. *felis*. This subspecies was later elevated to specific status by Goodey (1926).

#### Host range

Domestic cats (*Felis catus*) are the only confirmed natural host of *S. felis* (Speare, 1986). The species has been reported in cats from India (Chandler AC, 1925), Australia (Speare and Tinsley, 1986, 1987) and Thailand (Jitsamai, 2019). However, in the Thai case, the morphological identification was questionable, as it described a hexagonal stoma in the free-living female, a diagnostic feature characteristic of the parasitic female (Speare, 1986). Experimental infections of humans (*Homo sapiens*) and pigs (*Sus scrofa*) with *S. felis* did not result in patent infections (Speare, 1986).

### Strongyloides tumefaciens

#### Taxonomic history

In 1927, during the necropsy of a cat from Louisiana, United States (US), Price and Dikmans (1941) observed several tumour-like lesions in the large intestine. A similar case was reported in Florida 3 years later, with nodular masses also found in the colon of another cat. Microscopic examination in both cases revealed small nematodes embedded within the nodules, with no parasites detected elsewhere in the intestine. Recovery of intact specimens was hindered by the brittleness of formalin-fixed tissues; however, measurements from 2 incomplete worms indicated an estimated body length of 5000 µm, substantially larger than that of any known *Strongyloides* sp. in cats at the time (2370–3330 µm). Based on the relatively large size of the parasitic female and the presence of characteristic nodular colonic lesions, Price and Dikmans (1941) designated a new species, *Strongyloides tumefaciens*. No additional morphological descriptions of *S. tumefaciens* have since been published.

In 2019, Wulcan et al. (2019) reported similar nodular lesions in the colonic wall of *Strongyloides*-infected domestic cats from the Caribbean Island of St. Kitts. Worms recovered from these lesions were molecularly identified as *S. stercoralis* based on a 522 bp fragment of *cox*1. This finding prompted the authors to question the taxonomic validity of *S. tumefaciens* (Wulcan et al., 2019). However, in the absence of additional molecular or morphological analyses of either *S. tumefaciens* or *S. stercoralis* in feline hosts, the taxonomic status of *S. tumefaciens* remains unresolved.

#### Host range

Since its initial discovery, *S. tumefaciens* has been reported in domestic cats from the US (Malone et al., 1977; Lindsay et al., 1987) and Brazil (Moura et al., 2016), as well as in wild cats (*Felis chaus*) from India (Dubey and Pande, 1964). All of these reports relied on colonic pathology for species identification, a criterion later questioned by Wulcan et al. (2019).

### Strongyloides planiceps

#### Taxonomic history

*Strongyloides planiceps* was first identified by R.T. Leiper in rusty-spotted cats (*Prionailurus planiceps*) from Malaysia in 1927 (Rogers, 1939). Rogers (1939) described this species and initially named it *Strongyloides cati*. However, he later recognized that the name *S. cati* had already been used by Brumpt (1936) to describe a *Strongyloides* sp., now known as *S. felis*, from domestic cats in India. As Brumpt (1936)’s designation lacked a formal description, it was treated as a *nomen nudum* and held no official taxonomic standing. Nonetheless, to avoid confusion, Rogers (1943) renamed the species *S. planiceps*. Although *S. cati* (Rogers, 1939) remains the technically valid name under the rules of nomenclature, *S. planiceps* has become the widely accepted name in the scientific literature (Speare, 1986).

#### Host range

Natural infections with *S. planiceps* have been reported in raccoon dogs (*Nyctereutes procyonoides*) (Horie et al., 1981; Fukase et al., 1985; Sato et al., 2006), Japanese weasels (*Mustela itatsi*) (Fukase et al., 1985), red foxes (*Vulpes vulpes schrencki*) (Miyamoto and Inaoka, 1982), Japanese red foxes (*Vulpes vulpes japonica*) (Horie et al., 1989) and domestic cats (Horie et al., 1981; Fukase et al., 1983), most exclusively from Japan.

Natural infection in domestic dogs (*Canis lupus familiaris*) was suggested in 3 studies (Arizono, 1976; Horie et al., 1980; Fukase et al., 1984). Arizono (1976) described a strain of *S. planiceps* isolated from a dog in Kyoto, Japan, and maintained through serial passage in puppies; however, details of the original infection were not provided. Horie et al. (1980) experimentally infected cats with a *Strongyloides* sp. isolated from dogs and later detected larvated eggs in feline faeces, leading the authors to suspect the parasite was *S. planiceps*. The pre-inoculation infection status of the cats, however, was not assessed, leaving open the possibility of prior infection. Similarly, Fukase et al. (1984) recovered *Strongyloides* larvae from canine faeces and experimentally infected both dogs and cats. Larvated eggs were detected in the faeces of both hosts, and adult parasitic females recovered from the small intestine were morphologically identified as *S. planiceps*. Although this study strongly suggests that dogs can serve as suitable hosts, the identification was based on experimentally infected animals rather than naturally infected dogs. Therefore, the status of dogs as natural hosts of *S. planiceps* remains circumstantial and requires further confirmation.

Patent experimental infections of cats and dogs with *S. planiceps* derived from wild carnivores have been documented (Horie et al., 1981; Fukase et al., 1985). In the study by Horie et al. (1981), patency lasted from 0.9 to 3.6 years in cats and from 26 days to over 1 year in dogs. Fukase et al. (1985) did not report the duration of patency in experimentally infected cats.

## Morphology-based taxonomy of *Strongyloides*

### Development of morphological criteria

Efforts to establish morphological criteria for the taxonomy of *Strongyloides* spp. began in the early 20th century. Sandground (1925b) reviewed traits commonly used by early taxonomists to define new *Strongyloides* spp. and identified 2 reliable differentiating features: the stage passed in faeces and the ovary shape of the parasitic female. Chandler AC (1925) reclassified *Strongyloides* spp. into 2 groups, one represented by *S. stercoralis* and the other by *S. papillosus*. This framework was later criticized for being incomplete and overlapping (Desportes, 1944; Basir, 1950). A more comprehensive system was proposed by Little (1966a). He suggested that the ovary type and stomal shape of parasitic females, and the stage of progeny shed in faeces were the most important features for *Strongyloides* speciation. Using these criteria, Little characterized 6 established and 7 new *Strongyloides* spp. (Little, 1966a, b). However, he also acknowledged that these features alone might be insufficient to distinguish closely related species, such as the primate parasites *S. fuelleborni* and *S. cebus* (Little, 1966a).

Building upon Little (1966a)’s work, Speare (1986) refined *Strongyloides* taxonomy by incorporating additional distinguishing features across multiple life stages. These included post-vulval constriction and posterior vulval rotation in free-living females, as well as spicule morphology and peri-cloacal papillae arrangement in free-living males (Speare, 1986). These characteristics, along with those previously identified by Little (1966a) for parasitic females, were considered major morphological criteria for species delineation (Speare, 1986). Speare (1986) also proposed several minor traits, including body dimensions, host range and the presence of an autoinfective cycle, as supplementary identifiers. Together, these criteria form the basis of *Strongyloides* morphological taxonomy and remain widely used today in both research and clinical settings.

### Identification of the genus

#### Parasitic female

Parasitic females are slender and serpentine, measuring 1.5–10 mm in length and 27–95 µm in maximum width. The body is cylindrical, tapering slightly at the anterior end and abruptly at the tail. The thin body wall is covered by a finely striated cuticle. Tail is short and cone shaped. The head has a circumoral elevation but lacks lips, with a shallow, bilaterally symmetrical stoma. Cephalic papillae are indistinct, and amphids are positioned laterally. A single dome-shaped cervical papilla is present at the excretory pore level. The nerve ring crosses the oesophagus in the anterior quarter. The oesophagus is cylindrical, with a muscular anterior portion and a posterior part composed of 3 glandular nuclei (1 dorsal, 2 subventral). The intestine consists of 40 cells, each with a single nucleus, in 2 rows leading to a short rectum. The excretory system opens just posterior to the nerve ring. The reproductive system is didelphic, with equal, opposed uteri and reflexed ovaries, but lacks seminal receptacles. The vulva, situated approximately two-thirds of the body length from the anterior end, is a transverse slit with distinct margins. Eggs are arranged in a single row within the uterus. Paired nerve endings are present near the vulva, where the cuticle dorsal to the vulva is modified at its junction with the hypodermis (Little, 1966a, b; Speare, 1986; Grove, 1996; Castelletto et al., 2024).

*Strongyloides* spp. can be readily distinguished morphologically from other nematodes that may occur in the intestines of humans and animals by having only the parasitic female stage. Unlike most parasitic nematodes, *Strongyloides* spp. lack parasitic males, despite early reports suggesting otherwise (Faust EC, 1933). Parasitic females typically reside within the mucosal layer of the small intestine but may also be found freely in the intestinal lumen, particularly in cases of severe pathological reaction (Page et al., 2018). In humans and dogs, hyperinfection with *S. stercoralis* can lead to disseminated strongyloidiasis, where parasitic females have been identified in the mucosa of small bronchi and bronchioles (Higenbottam and Heard, 1976). In such severe cases, both parasitic females and eggs may be recovered from sputum and faeces (Bisoffi et al., 2013; Mati et al., 2014).

#### Free-living female

Free-living females are comparatively smaller (up to 1.5 mm in length) but broader (up to 85 µm at their widest point). They have a spindle-shaped body with a marked central enlargement to accommodate the egg-filled uterus, which occupies most of the body cavity. The head bears 2 lateral cephalic lobes, each containing small, inconspicuous papillae in subdorsal, lateral and subventral positions. Amphids are located posterior to the lateral papillae. The mouth is dorso-ventrally elongated, with a laterally compressed subglobular stoma bordered anteriorly by a collar-like cuticular structure. The rhabditoid oesophagus consists of a muscular corpus subdivided into anterior and posterior portions, a narrow isthmus and a terminal bulb with a well-developed valvular apparatus. The nerve ring encircles the oesophagus at the posterior end of the isthmus. The intestine comprises 22 cells arranged in dorsal and ventral rows. A short rectum leads to a subterminal anus, which has a slight lip-like swelling along its posterior edge. Phasmids are located laterally near the midpoint of the gradually tapering, finely pointed tail. The reproductive system is didelphic, with equal and opposed uteri and reflexed ovaries; the anterior branch runs along the right side of the intestine, and the posterior branch along the left. The vulva is positioned near mid-body and is associated with a short vagina; the terminal portion of the uterus serves as a seminal receptacle (Little, 1966a, b; Speare, 1986; Grove, 1996; Castelletto et al., 2024).

Free-living *Strongyloides* females must be carefully distinguished from free-living rhabditoid nematodes commonly found in soil, which can contaminate faecal cultures, particularly when samples are collected from bare ground. *Strongyloides* free-living females possess 2 broad lateral lips bearing 6 small papillae, whereas free-living rhabditoid nematodes typically have 4–6 lips with more prominent papillae. The buccal capsule in rhabditoids is longer and cylindrical with parallel sides, in contrast to the shorter and more compact buccal structure of *Strongyloides*. The oesophagus in *Strongyloides* is clearly divided into 4 regions, while in *Rhabditis*, the anterior muscular region is often reduced or absent, and the posterior corpus commonly expands into a muscular bulb, producing a characteristic mid-bulb morphology. Tail morphology also provides a useful differentiator; most free-living rhabditoid nematodes have a long, slender and whip-like tail, whereas the tail of *Strongyloides* is finely tapered and relatively short, typically not exceeding 15% of the total body length (Sandground, 1925a; Speare, 1986; Grove, 1996).

#### Free-living male

Free-living males are slightly smaller than females, measuring up to 1.2 mm in length and 55 µm at maximum width. The body wall, cuticle, head, oesophagus, intestine and excretory system are similar to those of the female. The reproductive system consists of a straight, tubular structure with an unreflexed testis extending from just posterior to the oesophagus to the mid-body. The seminal vesicle and vas deferens are poorly differentiated. The short cloaca houses a pair of equal, blade-like spicules with laterally bent, knob-like anterior ends and 2 supporting ribs that extend nearly to the tip. A thin membrane along the ventral edge gives the spicules a bow-like appearance. The gubernaculum is laterally compressed, with wing-like extensions forming a T-shaped posterior end in cross-section. The caudal papillae include a single unpaired papilla on the anterior cloacal lip and 6 bilaterally arranged papillae: 1 subventral preanal, 2 subventral adanal (anterior and posterior), 1 lateral postanal, 1 subventral postanal and 1 subdorsal postanal. The tail is shorter and broader than that of the female, ventrally curved when fixed and tapers abruptly to a point (Little, 1966a, b; Speare, 1986; Grove, 1996; Castelletto et al., 2024).

Several morphological features distinguish free-living *Strongyloides* males from rhabditoid males. In *Strongyloides*, the testis is broad, blunt-ended and unreflexed, whereas in rhabditoids, it is typically narrow and reflexed posteriorly. The spicules of rhabditoids are strongly curved, often appearing fused at the tip and lacking a ventral membrane. In contrast, *Strongyloides* spicules are less curved, possess a ventral membrane and are often extruded and diverge laterally in fixed specimens. The gubernaculum of *Strongyloides* includes a distinct medial plate separating the spicules, while in rhabditoids, the medial plate, if present, is typically small and does not extend between the spicules. Additionally, caudal alae are absent in *Strongyloides* but present in rhabditoids, where they typically bear 9 pairs of stalked papillae (Speare, 1986; Grove, 1996).

#### Egg

Eggs shed by parasitic and free-living females of *Strongyloides* are thin shelled and ellipsoid, with slightly flattened poles. They typically measure 40–55 µm in length, with a width ranging from one-half to three-quarters of the length. Each egg contains an underdeveloped rhabditiform larva at the time of passage (Little, 1966a, b; Speare, 1986; Grove, 1996).

**Figure 1.**
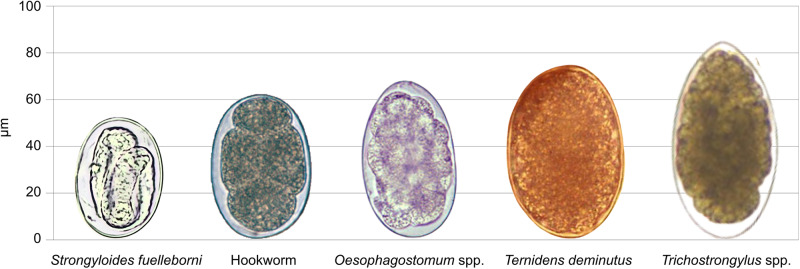
Exemplar eggs of selected intestinal nematodes, with size indicated in micrometres (µm). Figure 1 long description.

Careful differentiation is required in faecal samples to distinguish *Strongyloides* eggs from those of hookworms, *Ternidens deminutus, Oesophagostomum* spp. and trichostrongylids. *Strongyloides* eggs are generally smaller, have thinner shells and usually contain a developing larva rather than the 8- or 16-cell morula typical of these other species when freshly voided (Figure 1).

#### Rhabditiform larva

Four rhabditiform stages of *Strongyloides* spp. (L1r-L4r) have been identified. They are differentiated primarily by the increasing size following each moult (Little, 1966a, b; Speare, 1986; Grove, 1996; Castelletto et al., 2024).

The first-stage rhabditiform larva (L1r) of *Strongyloides* spp. measures 180–240 µm in length and 14–15 µm in width, with a mean length around 210 µm. The oesophagus comprises nearly one-third of the body length and is structurally similar to that of the free-living adult. The head has 2 cephalic lobes separated by a transversely elongated, oval mouth. The cylindrical stoma is 5–8 µm long, with a slightly thickened posterior wall. The nerve ring is initially located near the anterior end but migrates to the posterior isthmus prior to the first moult. The intestine consists of 22 uninucleate cells arranged in dorsal and ventral rows. The rectum is short, with the anus located 40–60 µm from the tail tip. A prominent, rhomboid-shaped genital primordium, containing 5–9 nuclei, lies ventrally near the mid-intestine. Although larval length nearly doubles before the first moult (depending on culture conditions), the oesophagus grows only slightly. There are no detectable morphological differences between L1r larvae originating from eggs of parasitic versus free-living females (Little, 1966a, b; Speare, 1986; Grove, 1996).

The L1r can be distinguished from other soil-transmitted nematodes by its shallow buccal cavity (4–8 µm), straight intestine and prominent lateral genital primordium. Hookworm larvae, in contrast, possess a smaller, more refractile genital primordium (<4 µm), while *Ternidens* spp. and *Rhabditis* spp. have longer tails and deeper buccal cavities (Schulte and Poinar, 1991; Bradbury, 2019; Buonfrate et al., 2022).

The second-stage rhabditiform larva (L2r) varies in morphology depending on whether development proceeds through the homogonic (direct) or heterogonic (indirect) life cycle. Larvae destined to become L3r (heterogonic development) undergo modest elongation and increased body width, measuring approximately 400 μm in length and 18–25 μm in width. In homogonic development, L2r transform into iL3 and exhibit more pronounced internal changes: the oesophagus elongates from 30% to 45% of body length, with the posterior portion becoming less muscular and more glandular, and oesophageal gland nuclei become more prominent. Intestinal cells (except the first and last pairs) divide, increasing the total from 22 to 40 nuclei. A notched tail of the emerging filariform larva forms within the cuticle in preparation for ecdysis. In both developmental pathways, the head is reorganized such that the cephalic lobes of the third-stage larva become lateral, in contrast to the dorsal and ventral positions observed in L1r and early L2r (Little, 1966a, b; Speare, 1986; Grove, 1996).

The third-stage rhabditiform larva (L3r) typically measures 550–700 μm in length and 22–30 μm in width. The oesophagus remains rhabditiform, increasing slightly in length and complexity, while the intestinal lumen widens. The genital primordium continues to expand, showing preliminary gonadal development. Morphological sexual differentiation begins to appear in L3r and becomes increasingly apparent as larvae approach L4r. The cuticle thickens, and head and tail structures mature; however, this stage remains non-infective and restricted to environmental development (Little, 1966a, b; Speare, 1986; Grove, 1996).

The fourth-stage rhabditiform larva (L4r) represents the final larval stage in the heterogonic cycle. These larvae typically measure 700–900 μm in length and 30–40 μm in width, with females slightly larger than males. The oesophagus adopts a structure resembling that of the free-living adult, including distinct corpus, isthmus and terminal bulb regions. Gonadal development progresses significantly: ovaries and testes are readily distinguishable and lie adjacent to the intestine. Though morphologically similar to free-living adults, L4r larvae are distinguishable by their smaller size and incomplete gonadal maturation. In females, the vulva is visible as a shallow, slit-like invagination but remains closed to the exterior (Castelletto et al., 2024). This stage precedes the final moult into sexually mature free-living adults (Little, 1966a; b; Speare, 1986; Grove, 1996).

#### Infective third-stage larva

In the homogonic (direct) life cycle, some L2r bypass the free-living phase and develop directly into infective third-stage filariform larvae (iL3) (Grove, 1996; Streit, 2008; Viney and Lok, 2015). The choice between homogonic and heterogonic development can be influenced by environmental factors such as temperature, humidity and host availability (Streit, 2008, 2017). In most *Strongyloides* spp., including *S. stercoralis* and *S. felis*, free-living development is limited to a single generation (Viney and Lok, 2015). However, under optimal culture conditions, some species may undergo multiple successive free-living generations. Hansen et al. (1969) reported 2–3 such generations in *S. fuelleborni*, comprising exclusively females that reproduced only in the presence of males from the first generation, while Premvati (1958a) observed only 1 generation in the same species. *S. planiceps* has been observed to complete up to 9 consecutive free-living generations (Yamada et al., 1991). iL3 represent the infective stage responsible for initiating new parasitic infections (Grove, 1996; Streit, 2008; Viney and Lok, 2015).

*Strongyloides* iL3 are slender and serpentine, measuring 400–700 µm in length and 12–20 µm in width. The cylindrical, filariform oesophagus constitutes approximately 40–45% of the total body length. The cuticle is finely striated, and the lateral alae are double, spaced about 4 µm apart, extending to the tail, where they form a distinctive notched tip. The head bears 2 inconspicuous lateral cephalic lobes with small subdorsal and subventral papillae, and a lateral amphid. The mouth is small and pore-like, with a shallow, laterally compressed stoma. The intestinal cells are arranged in dorsal and ventral rows, consisting of 40 nuclei, where the anterior and posterior pairs are uninucleate and the remainder binucleate. The excretory system resembles that of the free-living adult stages (Little, 1966a, b; Speare, 1986; Grove, 1996; Castelletto et al., 2024).

iL3 can be readily distinguished from other nematode larvae by their slender shape, elongated filariform oesophagus and, most notably, the notched tail tip, which is considered pathognomonic for the genus *Strongyloides*. No other nematodes exhibit this feature. Unlike hookworm iL3, *Strongyloides* iL3 are unsheathed, having shed the cuticle of the previous stage during ecdysis (Little, 1966a, b; Speare, 1986; Grove, 1996).

#### Autoinfective larva

*Strongyloides stercoralis* and *S. felis* are the only *Strongyloides* species for which an autoinfective cycle has been documented (Speare, 1986; Buonfrate et al., 2023). The third-stage autoinfective filariform larvae (L3a) share overall morphology and structural proportions with iL3 but are generally shorter and stouter. Their body length rarely exceeds 500 µm, with a width-to-length ratio of approximately 1:4 (Schad et al., 1993; Kim et al., 2005; Buonfrate et al., 2022, 2023).

Recently, *S. stercoralis* larvae exhibiting morphological features intermediate between L3a and parasitic females were observed in respiratory tract specimens from 2 human cases in Australia (Zhao et al., 2025b). These were identified as fourth-stage autoinfective filariform larvae (L4a) and are distinguished from L3a by their cone-shaped tail and a more developed genital primordium. The oesophagus remains filariform and occupies 37–46% of body length, a proportion intermediate between that of L3a and parasitic females. The genital rudiment is notably enlarged and contains a developing vulva enclosed within cuticular layers (Zhao et al., 2025b). Similar ‘juvenile’ parasitic females of *S. stercoralis* had been previously described in experimentally infected dogs (Faust EC, 1933) and marmosets (Mati et al., 2014), although Faust EC (1933)’s description may represent a mixture of parasitic and free-living forms (Zhao et al., 2025a). Detailed morphometric data for this stage remain unavailable.

### Identification of the species

Of the stages described above, the morphology of the parasitic female, free-living female and free-living male is particularly informative for differentiating *Strongyloides* spp. The distinguishing morphological and morphometric characteristics of these stages in *Strongyloides* spp. infecting humans and companion animals are discussed in the following sections and summarized in Tables 1 and 2.

**Table 1.** Comparative morphometrics of *Strongyloides* species infecting humans, dogs and cats Table 1 long description.

	*S. stercoralis*	*S. f. fuelleborni*	*S. f. kellyi*	*S. felis*	*S. tumefaciens*	*S. planiceps*
Egg size (µm)	60–75 × 30–40	50–60 × 30–40	51–55 × 34–37	nd	114–124 × 62–68	58–64 × 32–40
**Rhabditiform (L1) larvae**						
Body length (µm)	325–380	nd	nd	217–238	200	280
Body width* (µm)	17–20	nd	nd	nd	10	nd
Oesophagus length (µm)	89–94	nd	nd	nd	nd	nd
**Filariform (L3) larvae**						
Body length (µm)	490–630	560–680	572–654	525–615	nd	490–670
Body width* (µm)	15–16	14–17	15–17	14–16	nd	15
Oesophagus length (µm)	220–270	250–300	241–279	231–271	nd	259
Anus to tail (µm)	60–80	78–95	nd	nd	nd	69
**Parasitic female**						
Body length (µm)	2100–2700	2900–4200	2524–3476	2600–2920	5000	2370–3330
Body width* (µm)	30–40	43–55	42–54	39–45	109	40
Oesophagus length (µm)	480–670	710–980	637–931	690	750–1000	704
Mouth to vulva (µm)	1400–1800	1700–2700	1574–2208	1858	3400	1826
Anus to tail (µm)	40–70	45–70	44–54	63–104	106–114	38
**Free-living female**						
Body length (µm)	920–1700	1200–1300	1026–1156	1000–1200	nd	850–1480
Body width* (µm)	52–85	60–70	49–59	39–46	nd	50
Oesophagus length (µm)	125–150	140–155	133–155	143–171	nd	141
Mouth to vulva (µm)	470–820	570–680	492–564	nd	nd	nd
Anus to tail (µm)	80–170	137–160	114–136	94–110	nd	92
**Free-living males**						
Body length (µm)	810–1000	850–1100	692–896	895–985	nd	570–900
Body width* (µm)	40–50	38–52	nd	40–45	nd	38
Oesophagus length (µm)	110–125	120–130	111–127	138–152	nd	121
Anus to tail (µm)	55–95	80–100	72–92	63–79	nd	67
Spicule length (µm)	35–40	32–36	nd	32–36	nd	33–44
References	(Little, 1966a; Speare, 1986)	(Little, 1966a; Speare, 1986)	(Viney et al., 1991)	(Chandler AC, 1925; Speare and Tinsley, 1986)	(Price and Dikmans, 1941)	(Rogers, 1939)

nd, no data. *At the widest point.

**Table 2.** Distinguishing morphological features of *Strongyloides* species infecting humans, dogs and cats Table 2 long description.

	*S. stercoralis*	*S. f. fuelleborni*	*S. f. kellyi*	*S. felis*	*S. tumefaciens*	*S. planiceps*
Stages in fresh faeces	Rhabditiform larvae (L1r)	Larvated eggs	Larvated eggs	Rhabditiform larvae (L1r)	nd	Larvated eggs
**Parasitic female**						
Stomal shape (*en face* view)	Hexagonal	X-shaped	X-shaped	Rectangular	nd	Dumbbell with oesophageal teeth
Circumoral lobes	6	0	nd	6	nd	4 or 6
Ovary type	Straight	Spiral	Spiral	Straight	Straight	Spiral
Tail shape (lateral view)	Narrowly tapered	Bluntly rounded	Bluntly rounded	Narrowly tapered (more finely tapered, often pointed)	Narrowly tapered	Bluntly rounded
**Free-living female**						
Post-vulval narrowing	Absent or slightly narrowed	Present	Present (degree of narrowing intermediate between *S. stercoralis* and *S. f. fuelleborni*)	Present	nd	Absent
Posterior rotation of the vulva	Absent or slightly rotated (90°–100°)	Present (>100°)	Present (degree of rotation intermediate between *S. stercoralis* and *S. f. fuelleborni*)	Present (>100°)	nd	Absent
**Free-living males**						
Ventral border of spicule	Straight	Straight	nd	Straight	nd	Straight
Prominence of spicule ventral membrane	Inconspicuous	Prominent	Prominent	Inconspicuous	nd	Prominent
Distribution of caudal papillae	Subventral preanal and adanal papillae are longitudinally aligned.	Anterior adanal papillae positioned dorsally to the line connecting the subventral preanal and posterior adanal papillae.	Anterior adanal papillae positioned dorsally to the line connecting the subventral preanal and posterior adanal papillae.	Subventral preanal and adanal papillae are longitudinally aligned.	nd	Subventral preanal and adanal papillae are longitudinally aligned.
Other features	Presence of autoinfective larvae		Peri-vulval cuticle in the parasitic female; Phasmidial pore positioned midway between the post-cloacal papillae in the free-living male*	Presence of autoinfective larvae	Parasitic females found in the large intestine	
References	(Little, 1966a; Speare, 1986)	(Little, 1966a; Speare, 1986)	(Kelly et al., 1976; Speare, 1986; Viney et al., 1991)	(Chandler AC, 1925; Speare and Tinsley, 1986)	(Price and Dikmans, 1941)	(Arizono et al., 1976; Rogers, 1939; Sato et al., 2006; Speare, 1986)

L1, first-stage, rhabditiform larvae; NHP, non-human primate; nd, no data.

* Morphological features observed using scanning electron microscopy.

#### Parasitic female

**Figure 2.**
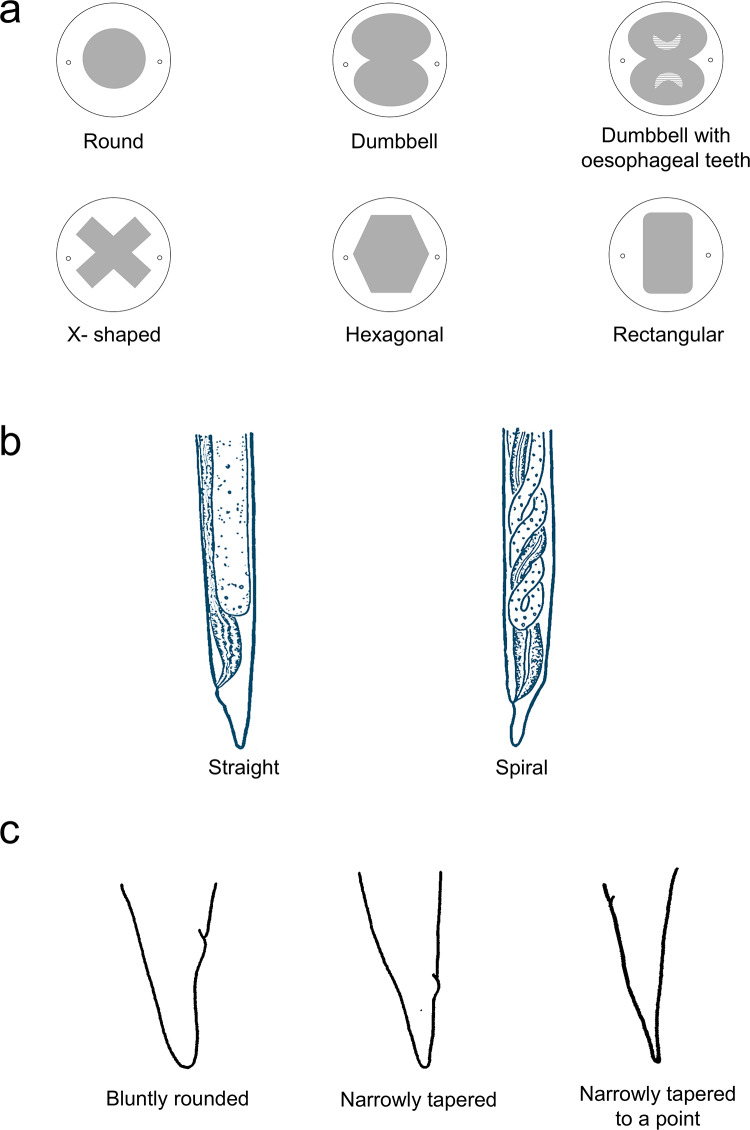
Distinguishing morphological features of parasitic females of *Strongyloides* spp. (a) Stomal shape in *en face* view; (b) ovary type, based on its orientation relative to the intestine; (c) tail morphology in lateral view, highlighting the degree of tapering. Modified from Speare (1986). See Table 2 for species-specific morphological features. Figure 2 long description.

**Figure 3.**
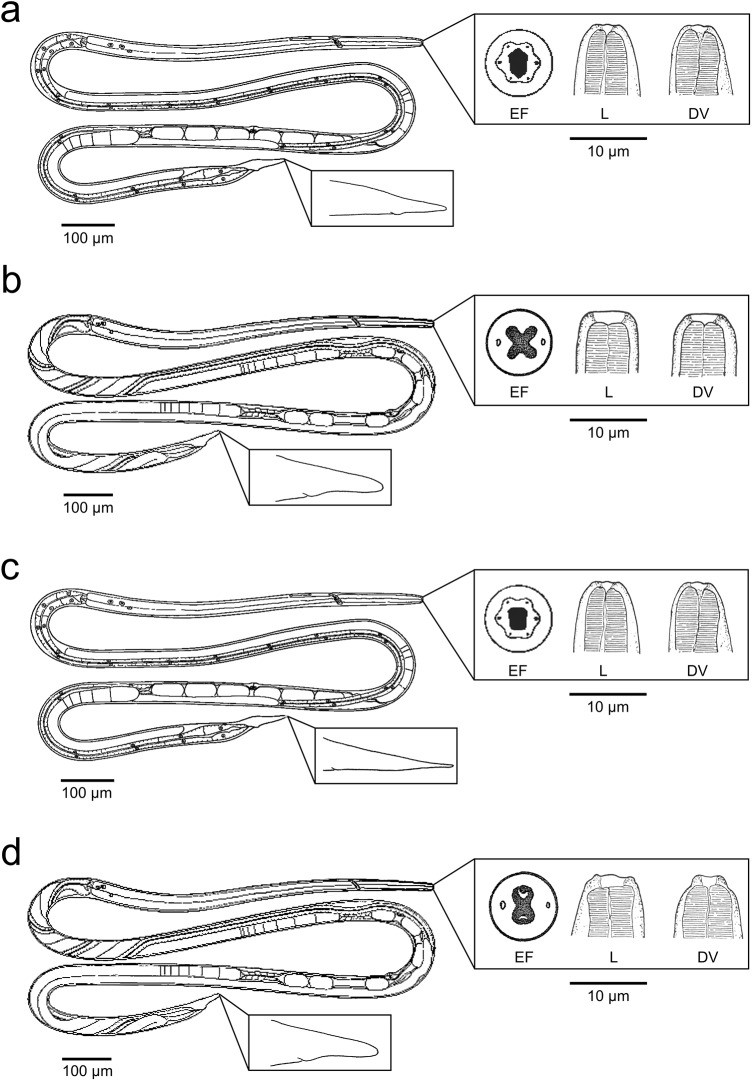
Parasitic females of *Strongyloides* spp.: (a) *Strongyloides stercoralis*, (b) *Strongyloides fuelleborni*, (c) *Strongyloides felis*, (d) *Strongyloides planiceps*. Each panel shows the full body of the nematode. Insets highlight the stoma and tail regions, with the stoma shown in *en face* (EF), lateral (L) and dorsoventral (DV) views, and the tail in lateral view. Modified from Little (1966a) and Speare (1986). Figure 3 long description.

Morphological features useful for species identification in the parasitic female include stomal shape (in *en face* view), the number and arrangement of lobes on the circumoral elevation, ovary configuration and tail morphology (Figures 2 and 3). Stomal shape can be categorized into 4 types: simple, angular, complex and those with oesophageal teeth (Speare, 1986). A simple stoma lacks angularity and may appear round, oval or dumbbell-shaped. Angular stomas include square, rectangular, hexagonal or badge-like configurations. Complex stomas are partitioned into multiple chambers with radiating subdivisions extending from a central cavity. A stoma with oesophageal teeth has anterior projections arising from the oesophagus and extending to the stomal margin (Figure 2A). While identifying the exact stomal shape may be technically challenging, classification into one of these categories is typically achievable and alone permits differentiation of several *Strongyloides* species (Speare, 1986; Sato et al., 2008).

The circumoral elevation is often divided into paired lobes, with species-specific variation in the number. In lateral or dorsoventral views, lobes may be absent or present in groups of 2, 4, 6 or 8. Species with 2 lobes typically have broad lateral lobes; those with 4 include lateral, dorsal and ventral lobes. In 6-lobed species, lobes are positioned laterally, subventrally and subdorsally, while 8-lobed species also have distinct dorsal and ventral lobes (Little, 1966a, b; Arizono et al., 1976). The prominence of these lobes varies among species, making enumeration less reliable in some cases. Although theoretically useful for differentiation, lobulation is generally considered a minor criterion, used only when other distinguishing features fail to separate species (Speare, 1986).

The didelphic ovary in parasitic females may be either straight (recurrent) or spiral. In spiral types, the ovaries follow the course of the intestine without encircling it. The anterior arm typically exhibits more pronounced spiralling than the posterior, and both arms maintain a consistent anatomical relationship with the intestine, spiralling uniformly in an anticlockwise direction from the anterior end (Figure 2B). Ovary type can aid taxonomic classification, but it is not a definitive diagnostic feature by itself. In species with spiral ovaries, females may reach sexual maturity before spiralling becomes morphologically apparent (Little, 1966a, b; Speare, 1986; Grove, 1996).

Tail morphology is another distinguishing feature among *Strongyloides* spp., with variation in the degree of taper and tip shape. Tails may range from narrowly tapered to bluntly rounded (Figure 2C). *S. felis* has a finely tapered, often pointed tail, whereas *S. stercoralis* typically has a less acutely tapered, blunt-ended tail (Speare, 1986). Although not exclusively diagnostic, tail morphology can support preliminary species differentiation, particularly between *S. felis* and *S. stercoralis* in feline hosts, pending confirmation through more definitive features, such as stomal shape (Speare, 1986).

#### Free-living female

**Figure 4.**
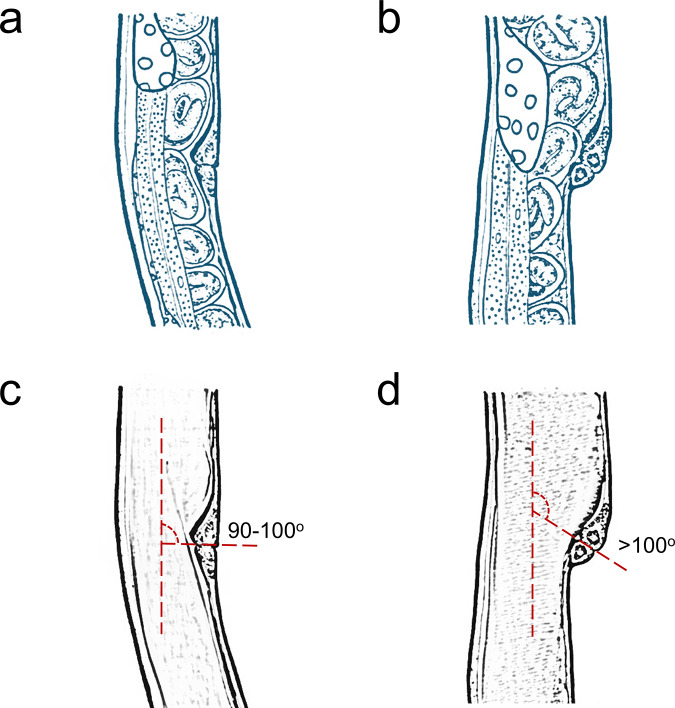
Vulval morphology in free-living females of *Strongyloides* spp. (a) Absence of post-vulval body narrowing (*S. stercoralis, S. planiceps*); (b) presence of post-vulval body narrowing (*S. fuelleborni, S. felis*); (c) absence of posterior vulval rotation (the vulval slit forms an angle of 90°–100° with the longitudinal body axis) (*S. stercoralis, S. planiceps*); (d) presence of posterior vulval rotation (the vulval slit forms an angle greater than 100° with the longitudinal body axis) (*S. fuelleborni, S. felis*). Modified from Speare (1986). Figure 4 long description.

The morphology of the free-living female *Strongyloides* spp. is largely conserved across species. Differentiation relies primarily on 2 peri-vulval features: post-vulval narrowing and vulval rotation. In some species, the body diameter remains relatively constant posterior to the vulva (Figure 4A), while in others, a reduction in diameter is observed (Figure 4B). *S. fuelleborni* and *S. felis* have pronounced post-vulval narrowing, whereas *S. stercoralis* shows a moderate reduction of approximately 15%. In species with less pronounced narrowing, the reduction is generally below 10% (Speare, 1986; Grove, 1996). A limitation of this trait is its environmental sensitivity; for instance, in *S. fuelleborni*, the degree of narrowing may vary with temperature (Premvati, 1958b).

By contrast, vulval rotation represents a more stable and taxonomically reliable feature. In most species, including *S. stercoralis* and *S. planiceps*, the vulval slit or short vagina forms an angle of 90°–100° with the longitudinal body axis (Figure 4C). However, in *S. fuelleborni* and *S. felis*, this angle exceeds 100°, producing a distinctly posteriorly rotated vulval appearance (Figure 4D). Unlike post-vulval narrowing, vulval rotation appears unaffected by environmental conditions and is considered a reliable criterion for species differentiation (Speare, 1986).

#### Free-living male

**Figure 5.**
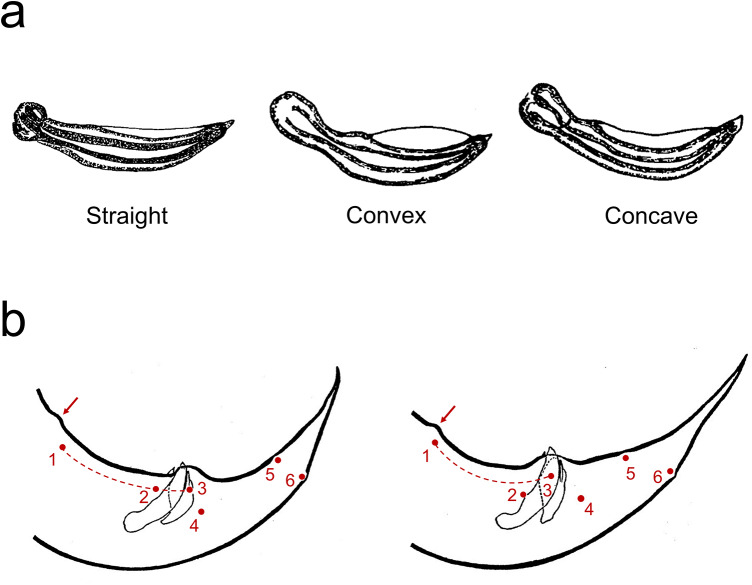
Distinguishing morphological features of free-living males of *Strongyloides* spp. (a) Spicule morphology, showing the shape of the ventral membrane; (b) tail showing the arrangement of caudal papillae, including (1) subventral preanal papilla, (2) anterior adanal papilla, (3) posterior adanal papilla, (4) lateral papilla, (5) subventral postanal papilla and (6) subdorsal postanal papilla. The subventral preanal papilla is aligned with the 2 adanal papillae in the left diagram but not in the right, as indicated by the red dashed line. The arrow indicates the preanal organ. Modified from Speare (1986). See Table 2 for species-specific morphological features. Figure 5 long description.

Taxonomically important features in the free-living male include spicule morphology, gubernaculum structure and the arrangement of caudal papillae. Most *Strongyloides* spp. possess sharply pointed spicule tips, though some species have blunted, hooked or laterally projected tips. Spicule curvature varies from straight to markedly curved; however, this trait has limited diagnostic value due to measurement variability and observer subjectivity (Speare, 1986). A more consistent taxonomic character is the shape of the spicule’s ventral membrane, which may appear convex, straight or concave (Figure 5A). Most species have a straight membrane, but its prominence varies, being more pronounced in *S. fuelleborni* compared to *S. stercoralis* (Speare, 1986; Grove, 1996).

The gubernaculum is relatively conserved across *Strongyloides* spp., except in *S. serpentis*, which displays a uniquely straight dorsal border. Minor differences in the dorsal pole and width-to-length ratio are observed among species but are not sufficient for definitive identification (Speare, 1986).

The arrangement of caudal papillae is among the most reliable taxonomic markers (Speare, 1986). Key distinguishing features include the position of the subventral preanal papilla relative to the preanal organ and the longitudinal alignment of the subventral preanal and adanal papillae (Speare, 1986). For instance, in *S. stercoralis*, the subventral preanal papilla is aligned with both adanal papillae, whereas in *S. fuelleborni*, the anterior adanal papilla is dorsally displaced (Figure 5B). Additionally, *S. stercoralis* has a more anteriorly located preanal organ relative to the cloacal opening, in contrast to its more posterior placement in *S. fuelleborni* (Speare, 1986).

The position of the phasmidial pore in the free-living male is particularly informative in distinguishing subspecies of *S. fuelleborni*. In *S. f. kellyi*, the phasmidial pore lies midway between the 2 post-cloacal papillae, while in *S. f. fuelleborni*, it is situated adjacent to the anterior-most cloacal papilla (Viney et al., 1991). Owing to their minute size, phasmidial pores require visualization using scanning electron microscopy.

#### Other distinguishing features

The developmental stage present in freshly voided faeces can assist in preliminary species identification. While all *Strongyloides* spp. are oviparous, hatching may occur internally, resulting in the passage of L1r in faeces, as observed in *S. stercoralis* and *S. felis*, or externally, with eggs passed in faeces and hatching occurring in the environment, as seen in *S. fuelleborni* and *S. planiceps*. Some other animal-infecting *Strongyloides* spp., such as *S. ratti*, may shed both eggs and L1r in faeces (Speare, 1986; Viney and Lok, 2015).

However, faecal stages alone are unreliable for definitive species identification, particularly when examination is delayed. For example, eggs of *S. f. fuelleborni* may hatch into L1r within 6–10 h at 23–25 °C and within 2–7 h at 37 °C (Cordi and Otto, 1934). Infection intensity may also influence which stage is excreted. Although rare, eggs of *S. stercoralis* have been documented in freshly voided faeces during hyperinfection (Bisoffi et al., 2013; Mati et al., 2014).

The detection of *Strongyloides* L1r, parasitic females or eggs in respiratory or urinary tract specimens is indicative of disseminated *S. stercoralis* infection (Buonfrate et al., 2022). *S. stercoralis* is currently the only species known to cause dissemination in natural infections (Buonfrate et al., 2023).

### Challenges in morphology-based taxonomy

Reliance on morphology-based taxonomic tools to identify and differentiate *Strongyloides* spp. presents several challenges. Firstly, accurate identification requires experienced morphologists capable of recognizing subtle diagnostic characters, a skill set that is becoming increasingly scarce as formal training in parasitological morphology declines (Bradbury et al., 2022). For example, distinguishing the 2 larvae-shedding species, *S. stercoralis* and *S. felis*, requires detailed assessment of minute differences in stomal and tail morphology in parasitic females and vulval configuration in free-living females (Speare, 1986).

Secondly, the life cycle stages most informative for speciation, parasitic females and free-living adult males and females, are difficult to obtain. Parasitic females can be recovered from animals by necropsy or intestinal biopsy but are rarely accessible from humans. Recovery from faeces is occasionally possible following anthelmintic treatment; however, these worms are dead and often show degenerate morphological changes that limit reliable identification (Speare, 1986). Speare (1986) noted postmortem pigment accumulation and ovarian vacuolation in parasitic females, which can obscure determination of ovary type, a key taxonomic character. Free-living adults are facultative and require prolonged culture (≥5 days) to obtain (Buonfrate et al., 2022). Morphology of free-living females can be influenced by culture temperature: *S. fuelleborni* has been reported to show markedly reduced post-vulval narrowing when cultured above 30 °C or below 25 °C (Premvati, 1958b), although this was not observed in *S. felis* (Speare, 1986). Easily recoverable faecal stages (e.g. eggs or L1r) are morphologically uniform across *Strongyloides* spp., limiting their taxonomic utility (Speare, 1986).

Thirdly, although morphometrics can help distinguish *Strongyloides* from other nematodes, they are of limited value for differentiating species within the genus (Table 1). Specimen measurements may be influenced by artefacts introduced during processing prior to microscopic examination. For example, compression under coverslips can flatten cylindrical worms, artificially increasing width, whereas chemical fixation may cause shrinkage or alter proportions (Speare, 1986). Speare (1986) found that preservation in 70% ethanol for 48 h reduced the length of *Strongyloides* parasitic females by approximately 15% and iL3 by 13.5%, while 10% formalin caused comparatively less shrinkage (7.5% for iL3).

Finally, while established morphological criteria allow for the differentiation of many *Strongyloides* spp., they do not reliably distinguish closely related species or resolve interspecific relationships. Chandler AC (1925) proposed the earliest framework for grouping *Strongyloides* spp., though he erroneously assigned several species at the subspecific level. His classification divided species into 2 groups: the *S. stercoralis* group and the *S. papillosus* group. The *S. stercoralis* group is characterized by parasitic females with straight ovaries, a simple stoma, a narrowly tapered tail and the shedding of L1r in faeces. Free-living males in this group possess pointed spicules and have the subventral preanal and adanal papillae aligned longitudinally. Members include *S. stercoralis, S. felis* and *S. procyonis*. In contrast, the *S. papillosus* group includes species with parasitic females that have spiralled ovaries, a complex stoma and shed eggs in faeces. Free-living males also have pointed spicules, but the first adanal papilla is displaced dorsally relative to the line connecting the subventral preanal and second adanal papilla. This group includes *S. papillosus, S. fuelleborni, S. cebus, S. planiceps, S. ransomi* and *S. venezuelensis*. While Chandler AC (1925)’s framework offers a broad morphological classification that partially aligns with later molecular-genetic findings, it is overly simplistic and does not account for all known species.

## Molecular taxonomy of *Strongyloides*

### Molecular taxonomic tools and techniques

The advent of molecular techniques and DNA sequencing has revolutionized the taxonomy of *Strongyloides* spp. Commonly used genetic markers for characterizing *Strongyloides* include the small subunit (18S) rRNA gene, mitochondrial cytochrome c oxidase subunit I (*cox*1), the large subunit (28S) rRNA gene and the Internal Transcribed Spacer (ITS) region (Bradbury et al., 2021; Al-Jawabreh et al., 2024b). Of these, partial *cox*1 sequences (217–750 bp) and single nucleotide polymorphisms within the hypervariable region IV (HVR-IV; 23-39 bp) of *18S rRNA* are particularly informative for inferring species identity and intraspecific relationships (Hasegawa et al., 2009, 2010; Bradbury et al., 2021). These markers have been widely adopted in phylogenetic and population genetic studies of the genus (Table 3).

**Table 3.** Summary of phylogenetic or population genetic studies of *Strongyloides* species infecting humans, dogs and cats Table 3 long description.

Studies	Country	Host species	DNA source	Gene region(s)	Sequencing methods	GenBank accessions
***Strongyloides stercoralis***						
Dorris et al., 2002	USA	*Canis lupus familiaris*	iL3	*18S rRNA* (1200 bp)	Sanger	AF279916; AJ417023
Nilforoushan et al., 2007	Iran	*Homo sapiens*	iL3	ITS-1 (680 bp)	Sanger	nd
Kousha et al., 2009	Iran	*Homo sapiens*	iL3	*18S rRNA* (975 bp)	Sanger	EF062571
Hasegawa et al., 2009	Japan	*Canis lupus familiaris*	iL3, parasitic females	*18S rRNA* HVRs	Sanger	AB453316
	Japan, Philippine	*Homo sapiens*	iL3, L1r	*18S rRNA* HVRs	Sanger	AB453315
Hasegawa et al., 2010	Japan	*Canis lupus familiaris*	iL3, parasitic females	*cox1* (722 bp)	Sanger	AB526302; AB526303
	Japan, Tanzania	*Homo sapiens*	iL3	*cox1* (722 bp)	Sanger	AB526297–AB526301
Labes et al., 2011	Indonesia	*Pongo pygmaeus*^ϯ^	L1r, iL3	*18S rRNA* (134–400 bp), ITS-1 (202 bp)	Sanger	JF699139–JF699167
Schär et al., 2014	Cambodia	*Homo sapiens*	L1r	*18S rRNA* HVR-I and HVR-IV	Sanger	KF926658–KF926662
Kikuchi et al., 2016	Myanmar, Japan	*Homo sapiens*	iL3	Whole genome	Illumina	PRJDB5112
Laymanivong et al., 2016	Lao	*Homo sapiens*	Free-living males	*cox1* (790 bp), *18S rRNA* (568 bp)	Sanger	*cox1*: KU962139–KU962178 *18S rRNA*: KU962179–KU962182
Hasegawa et al., 2016	Central African Republic	*Homo sapiens*	iL3	*cox1* (714 bp), *18S rRNA* HVR-IV	Sanger	*cox1*: LC085498–LC085500 *18S rRNA* HVR-IV: LC085481–LC085483
Nagayasu et al., 2017	Myanmar, Thailand, Japan, Uganda	*Homo sapiens*	iL3, free-living adults	*cox1* (711 bp), *18S rRNA* (333 bp), *28S rRNA* (733–736 bp)	Sanger	*cox1*: LC179022–LC179542 *28S rRNA*: LC259531–LC259841
	Myanmar, Japan	*Canis lupus familiaris*	iL3, free-living adults	*cox1* (711 bp), *18S rRNA* (333 bp), *28S rRNA* (733–736 bp)	Sanger	
Jaleta et al., 2017	Cambodia	*Homo sapiens, Canis lupus familiaris*	iL3, L1r, free-living adults	*cox1* (650 bp), *18S rRNA* HVR-I and HVR-IV	Sanger	KU724124–KU724129; KX226367–KX226384; KY548505
			Free-living males	Whole genome	Illumina	PRJEB20999
Thanchomnang et al., 2017	Thailand	*Homo sapiens*	Free-living adults	*cox1* (696 bp), *18S rRNA* (494 bp)	Sanger	*cox1*: KY081224–KY081232; KY081234–KY081242 *18S rRNA*: KY081221; KY081223
Gorgani-Firouzjaee et al., 2018	Iran	*Homo sapiens*	iL3, total serum DNA	*cox1 (650 bp), 18S rRNA* (101 bp)	Sanger	*cox1:* MG995852
Zhou et al., 2019	China	*Homo sapiens*	iL3, free-living adults	*cox1* (552 bp), *18S rRNA* HVR-I and HVR-IV, ITS	Sanger	*cox1*: MK040537–MK040539
			iL3, free-living males and females	Whole genome	Illumina	PRJNA517237
Barratt et al., 2019	Cambodia, Laos, Angola, Ethiopia, Senegal, USA, Brazil, Nigeria, Guinea, Ethiopia, Australia, India, Italy, Ivory Coast	*Homo sapiens*	Faecal DNA	*cox1* (217 bp), *18S rRNA* HVR-I (∼434 bp) and HVR-IV (∼255 bp)	Illumina	*cox1*: MN076433–MN076452; MN076454–MN076464 *18S rRNA* HVR-IV: MN076387–MN076414; MN076416–MN076423 *18S rRNA* HVR-I: MN076328–MN076360; MN076371–MN076380
	Italy, Cambodia, USA	*Canis lupus familiaris*	Faecal DNA	*cox1* (217 bp), *18S rRNA* HVR-I (∼434 bp) and HVR-IV (∼255 bp)	Illumina	*cox1*: MN076424–MN076432 *18S rRNA* HVR-IV: MN076381–MN076386 *18S rRNA* HVR-I: MN076327; MN076361–MN076370
Beknazarova et al., 2019	Australia	*Homo sapiens, Canis lupus familiaris*	Faecal DNA	*cox1* (217 bp), *18S rRNA* HVR-I (∼434 bp) and HVR-IV (∼255 bp)	Illumina	*cox1*: MK434217–MK434258 *18S rRNA:* MK468654–MK468677
Basso et al., 2019	Switzerland	*Canis lupus familiaris*	L1r	*cox1* (688 bp), *18S rRNA* HVR-I (863 bp) and HVR-IV (712 bp)	Sanger	*cox1*: MH932101–MH932103 *18S rRNA* HVR-IV: MH932095–MH932097 *18S rRNA* HVR-I: MH932098–MH932100
Fadaei Tehrani et al., 2019	Iran	*Homo sapiens*	iL3, free-living adults	*cox1* (509 bp)	Sanger	MG251317–MG251326
Wulcan et al., 2019	St. Kitts	*Felis catus*	Tissue DNA (colonic nodules)	*cox1* (522 bp)	Sanger	MK463927; MK463928
Sanpool et al., 2020	Thailand	*Homo sapiens, Canis lupus familiaris*	Free-living males	*cox1* (552 bp), *18S rRNA* HVR-IV (331 bp)	Sanger	*cox1*: MN509454–MN509463 *18S rRNA* HVR-IV: MN498129–MN498130
Obanda et al., 2019	Kenya	*Papio cynocephalus*	iL3	ITS-1 (300–338 bp)	Sanger	KT307984–KT307989
Aupalee et al., 2020	Thailand	*Homo sapiens*	L1r, iL3	*cox1* (552 bp), *18S rRNA* HVR-I and HVR-IV	Sanger	*cox1:* MN871994–MN872230
				Whole genome	Illumina	PRJNA602131
Ko et al., 2020	Myanmar, Japan, Peru,	*Homo sapiens, Canis lupus familiaris* (Myanmar)	iL3, free-living adults	*18S rRNA, 28S rRNA*	Sanger	*18S rRNA:* LC537177–LC537179 *28S rRNA:* LC537198–LC537200
				Mitochondrial genome	Illumina	LC533901–LC533903; LC533830
Barratt and Sapp, 2020*	na	*Homo sapiens, Canis lupus familiaris, Felis catus*	na	*cox1* (217 bp), *18S rRNA* HVR-I (∼434 bp) and HVR-IV (∼255 bp)	na	MT436714
Bae et al., 2020	Korea	*Homo sapiens*	Tissue DNA (colon and duodenum biopsies)	*cox1* (509 bp)	Sanger	MT932865
Repetto et al., 2022	Argentina	*Homo sapiens*	Faecal DNA	*cox1* (404 bp)	Sanger	MW680430–MW680476
Salant et al., 2021	Israel	*Canis lupus familiaris*	Faecal DNA	*cox1* (217 bp), *18S rRNA* HVR-I (∼434 bp) and HVR-IV (∼255 bp)	Sanger	*cox1*: MW857116 *18S rRNA* HVR-IV: MW857133 *18S rRNA* HVR-I: MW857130
Borrás et al., 2023	Argentina	*Canis lupus familiaris*	Faecal DNA	*cox1* (404 bp)	Sanger	*cox1:* OQ921397
Nosková et al., 2023	Czech Republic; Slovakia	*Pongo pygmaeus*^ϯ^; *Pongo abelii*^ϯ^	L1r	*cox1* (263–627 bp), *18S rRNA* HVR-I (391–457 bp) and HVR-IV (235–281 bp)	Sanger	*cox1:* OM392049–OM392052 *18S rRNA:* OM423625–OM423632
Nosková et al., 2024	Czech Republic	*Canis lupus familiaris*	Faecal DNA	*cox1* (628 bp), *18S rRNA* HVR-I (466 bp) and HVR-IV (290 bp)	Sanger and Illumina	*cox1:* OR587862
Beiromvand et al., 2024	Iran	*Homo sapiens*	iL3, free-living adults	*cox1* (552 bp), *18S rRNA* HVR-I and HVR-IV	Sanger	*cox1*: LC772965–LC772977
				Whole genome	Illumina	PRJEB64686
de Ree et al., 2024	Bangladesh	*Homo sapiens, Canis lupus familiaris*	iL3, free-living adults	*cox1* (552 bp), *18S rRNA* HVR-I and HVR-IV	Sanger	*cox1*: OR804688–OR804712; OR809277–OR809299
				Whole genome	Illumina	PRJEB70604
Kirkwood and Šlapeta 2024	Australia	*Canis lupus familiaris*	Faecal DNA	*cox1* (217 bp), *18S rRNA* HVR-I (∼434 bp) and HVR-IV (∼255 bp)	Illumina	PRJNA1047805
Dalimi and Jaffarian, 2024	Iran	*Homo sapiens*	Faecal DNA	*cox1* (509 bp)	Sanger	MH921561–MH921564
Semnan et al., 2024	Iran	*Homo sapiens*	iL3	*cox1* (509 bp)	Sanger	OR552505–OR552514
Liu et al., 2025	Bangladesh, Cambodia, Thailand, Fiji	*Homo sapiens, Canis lupus familiaris*	iL3	Whole genome	Illumina	PRJEB80566
***Strongyloides fuelleborni fuelleborni***						
Dorris et al., 2002	nd (Africa)	nd (NHP)	Formalin-fixed material	*18S rRNA* (332 bp)	Sanger	AJ417030
Hasegawa et al., 2009	Tanzania	*Homo sapiens*	iL3	*18S rRNA* HVRs	Sanger	AB453320
	Gabon	*Pan troglodytes, Gorilla gorilla*	iL3	*18S rRNA* HVRs	Sanger	AB453321, AB453322
	Japan	*Macaca fuscata*	iL3	*18S rRNA* HVRs	Sanger	AB453317–AB453319; AB453335
Hasegawa et al., 2010	Tanzania	*Homo sapiens, Papio cynocephalus, Pan troglodytes*	iL3	*cox1* (722 bp)	Sanger	AB526282–AB526286; AB526306
	Gabon	*Pan troglodytes, Gorilla gorilla*	iL3	*cox1* (722 bp)	Sanger	AB526287–AB526289
	Japan	*Macaca fuscata*	iL3	*cox1* (722 bp)	Sanger	AB526290–AB526294
Labes et al., 2011	Indonesia	*Homo sapiens, Pongo pygmaeus*	L1r, iL3	*18S rRNA* (134–400 bp), ITS-1 (202 bp)	Sanger	JF699139–JF699167
Makouloutou et al., 2014	Gabon	*Gorilla gorilla, Pan troglodytes*	iL3	*18S rRNA* (1142 bp)	Sanger	AB821045, AB821046
Hasegawa et al., 2016	Central African Republic	*Homo sapiens, Gorilla gorilla, Pan troglodytes*	iL3	*cox1* (714 bp), *18S rRNA* HVR-IV	Sanger	*cox1*: LC085501–LC085509 *18S rRNA* HVR-IV: LC085484–LC085490
Thanchomnang et al., 2017	Thailand	*Homo sapiens*	Free-living adults	*cox1* (696 bp), *18S rRNA* (494 bp)	Sanger	*cox1*: KY081233 *18S rRNA*: KY081222
Frias et al., 2018	Malaysia	*Pongo pygmaeus, Nasalis larvatus, Trachypithecus cristatus, Macaca fascicularis*	iL3	*cox1* (716 bp)	Sanger	LC197964–LC198003; LC317016–LC317043
Thanchomnang et al., 2019	Thailand, Lao	*Macaca fascicularis*	Free-living adult males	*cox1* (646 bp), *18S rRNA* (356 bp and 219 bp)	Sanger	*cox1*: MH049697–MH049729 *18S rRNA*: MH045486–MH045488
Obanda et al., 2019	Kenya	*Papio cynocephalus*	iL3	*cox1* (542–682 bp)	Sanger	KT381715–KT381719
Barratt et al., 2019	India, Guinea-Bissau, Senegal	*Homo sapiens*	Faecal DNA	*cox1* (217 bp), *18S rRNA* HVR-IV (∼255 bp)	Illumina	*cox1*: MN076453, MN076442, *18S rRNA* HVR-IV: MN076415, MN076407, MN076408, *18S rRNA* HVR-I: MN076378, MN076379
Potters et al., 2020	Democratic Republic of the Congo	*Homo sapiens*	nd	*18S rRNA* (271 bp)	Sanger	nd
Janwan et al., 2020	Thailand	*Homo sapiens, Macaca nemestrina*	Free-living adult males	*cox1* (710 bp), *18S rRNA* HVR-IV (526 bp)	Sanger	*cox1*: MT479191–MT479214, *18S rRNA:* MT484268; MT484269
Ko et al., 2023	Myanmar, Japan	*Macaca mulatta, Macaca fuscata, Trachypithecus francoisi*^ϯ^*, Pygathrix nemaeus*^ϯ^*, Pongo pygmaeus*^ϯ^*, Symphalangus syndactylus*^ϯ^*, Saimiri boliviensis*^ϯ^	iL3, free-living adults	*cox1* (710 bp), *18S rRNA* (485 bp)	Sanger	*cox1*: OM570560–OM570572; OM570197–OM570221
				Mitochondrial genome	Illumina	OL505577; OL672153; OL672152; OL692833; OL890689; OL672246; OL739736
Richins et al., 2023	St. Kitts	*Chlorocebus aethiops*	Faecal DNA	*cox1* (217 bp), *18S rRNA* HVR-I (∼434 bp) and HVR-IV (∼255 bp)	Illumina	*cox1:* OQ190184–OQ190189, *18S rRNA* HVR-IV: OQ190398–OQ190403, *18S rRNA* HVR-I: OQ190413–OQ190415
Juhasz et al., 2023	UK	*Papio anubis*^ϯ^	iL3	*18S rRNA* (435 bp)	Sanger	OR395366; OR395367
de Ree et al., 2024	Bangladesh	*Homo sapiens*	iL3, free-living adults	*cox1* (552 bp), *18S rRNA* HVR-I and HVR-IV	Sanger	*cox1*: OR805174–OR805181
				Whole genome	Illumina	PRJEB70604
Juhász et al., 2025	Malawi	*Chlorocebus pygerythrus*	iL3	*cox1* (272 bp)	Sanger	PQ846734–PQ846739
Richins et al., 2025	UK	*Papio anubis*^ϯ^	iL3, free-living adults	Whole genome, mitochondrial genome, *cox1* (288 bp), *18S rRNA* (216 bp)	Illumina	*cox1*: PV555680; PV555681, *18S rRNA*: PV558842, Mitochondrial genome: PV564803–PV564809, Whole genome: PRJNA1254725
Zhao et al., 2026	Gabon	*Homo sapiens*	Faecal DNA	*cox1* (217 bp), *18S rRNA* HVR-IV (255–258 bp)	Illumina	*cox1*: PV716215–PV716218, *18S rRNA* HVR-IV: PV716221–PV716223
Zhao et al., 2026	Papua New Guinea	*Homo sapiens*	Faecal DNA	*cox1* (217 bp), *18S rRNA* HVR-I (∼432 bp) and HVR-IV (∼252 bp)	Illumina	*cox1*: PQ774615–PQ774617, *18S rRNA* HVR-I: PV489783, *18S rRNA* HVR-IV: PQ774622
***Strongyloides fuelleborni kellyi***						
Dorris et al., 2002^β^	Papua New Guinea	*Homo sapiens*	Formalin-fixed material	*18S rRNA* (330 bp)	Sanger	AJ417029
***Strongyloides planiceps***						
Hasegawa et al., 2009	Japan	*Nyctereutes procyonoides*	Parasitic females	*18S rRNA* HVRs	Sanger	AB453324; AB453331
Hasegawa et al., 2010	Japan	*Nyctereutes procyonoides*	Parasitic females	*cox1* (722 bp)	Sanger	AB526295; AB526296
Ko et al., 2020	Japan	*Nyctereutes procyonoides*	Parasitic females	*cox1* (711 bp), *18S rRNA, 28S rRNA*	Sanger	*cox1:* LC536594–LC536600, *18S rRNA:* LC536168, LC537182 *28S rRNA:* LC537191
***Strongyloides* spp. of unconfirmed identity**						
Jitsamai, 2019	Thailand	*Felis catus*	iL3, free-living adults	*18S rRNA* (708 bp)	nd	nd
Ko et al., 2020	Myanmar	*Felis catus*	iL3, free-living adults	*cox1* (711 bp), *18S rRNA, 28S rRNA*	Sanger	*cox1:* LC536433–LC536460, *18S rRNA:* LC536162, LC537183, *28S rRNA:* LC537192
				Mitochondrial genome	Illumina	LC533411
Beknazarova et al., 2019	Australia	*Canis lupus familiaris*	Faecal DNA	*cox1* (217 bp), *18S rRNA* HVR-I (∼434 bp) and HVR-IV (∼255 bp)	Illumina	*cox1*: MK434220–MK434222; MK434226; MK434227, *18S rRNA:* MK468661; MK468675
Zhao et al., 2026	Papua New Guinea	*Homo sapiens*	Faecal DNA	*18S rRNA* HVR-I (432 bp) and HVR-IV (∼252 bp)	Illumina	*18S rRNA* HVR-I: PV489780–PV489782, *18S rRNA* HVR-IV: PQ774624

na, not applicable; nd, no data; NHP, non-human primate; iL3, infective third-stage filariform larvae; L1r, rhabditiform larvae.

^ϯ^Zoo-kept animal; ^β^Species identity questionable.

* Secondary analysis of existing data.

More recently, mitochondrial genome and whole-genome sequencing have been applied in *Strongyloides* taxonomy (Ko et al., 2023; de Ree et al., 2024; Liu et al., 2025; Richins et al., 2025). Compared to traditional markers such as *cox*1 and *18S rRNA* HVR-IV, these approaches offer a larger number of phylogenetically informative sites, enabling higher resolution at both interspecific and intraspecific levels. However, their use is limited by the scarcity of high-quality reference genomes. To date, genome assemblies are available for only 4 species: *S. stercoralis, S. papillosus, S. ratti* and *S. venezuelensis* (Hunt et al., 2016; Kounosu et al., 2024). Most assemblies are fragmented, having been generated using short-read sequencing technologies, although long-read data have recently improved genome quality for *S. stercoralis* and *S. ratti* (Kounosu et al., 2024). The reference genome for *S. stercoralis* (strain PV001) is derived from a laboratory-maintained line originally isolated from a natural infection over 4 decades ago (the UPD strain), which may not accurately represent the genetic diversity of contemporary wild populations (Schad et al., 1984; Hunt et al., 2016; Kounosu et al., 2024).

Conventional polymerase chain reaction (PCR) followed by Sanger sequencing is suitable for barcoding individual *Strongyloides* isolates. However, when analysing total DNA extracted from complex biological samples such as faeces, where multiple *Strongyloides* genotypes may be present, deep amplicon sequencing is required. A metabarcoding assay targeting regions of *cox*1 and *18S rRNA* HVR-I and HVR-IV has been developed for use on the Illumina MiSeq platform (Barratt et al., 2019) and applied in several faecal surveys (Table 3). However, such metabarcoding approaches face several challenges, including the need for extensive protocol optimization. For example, sequencing success rates from faecal DNA extracts were reported at 63% for *cox*1, 80% for *18S rRNA* HVR-I and 68% for HVR-IV (Barratt et al., 2019). In addition, these analyses require substantial bioinformatics expertise, and incomplete reference libraries may further impact the accuracy and resolution of taxonomic assignments.

### Intraspecific and cryptic diversity

#### Strongyloides stercoralis

Genotyping of *Strongyloides* was first attempted by Ramachandran et al. (1997) using PCR–restriction fragment length polymorphism (RFLP). This study analysed partial *28S rRNA* and ITS across multiple *Strongyloides* spp., including *S. stercoralis*. While all human-derived *S. stercoralis* isolates were indistinguishable, several distinct RFLP profiles differentiated them from a canine strain originally isolated from a naturally infected beagle and subsequently maintained in laboratory dogs at the University of Pennsylvania (the UPD strain) (Schad et al., 1984). To identify more informative markers for species discrimination, Hasegawa and colleagues examined HVR-I to HVR-IV of *18S rRNA* and a 722 bp fragment of *cox1*. Although species-specific clustering was observed, these loci did not consistently distinguish *S. stercoralis* isolates from humans, dogs and chimpanzees (Hasegawa et al., 2009, 2010).

Two subsequent genotyping surveys in Asia demonstrated the phylogenetic divergence of *S. stercoralis* into 2 lineages based on *cox1* sequence data: one infecting both humans and dogs (*cox1* lineage A) and the other found exclusively in dogs (*cox1* lineage B) (Jaleta et al., 2017; Nagayasu et al., 2017). Jaleta et al. (2017) also analysed the *18S rRNA* HVR-I and HVR-IV regions, finding that *18S rRNA* HVR-IV haplotype A corresponded with *cox1* lineage A, while *18S rRNA* HVR-IV haplotype B corresponded with *cox1* lineage B. Furthermore, they observed that although *cox1* and *18S rRNA* HVR-IV sequences reliably distinguished the 2 lineages, *18S rRNA* HVR-I haplotypes did not correlate with host specificity.

The genotyping scheme established by Jaleta et al. (2017) and Nagayasu et al. (2017) has since been expanded in multiple population genetic studies. *cox*1 lineage A has been identified globally in humans, dogs, cats and NHPs (Hasegawa et al., 2010; Laymanivong et al., 2016; Jaleta et al., 2017; Thanchomnang et al., 2017; Barratt et al., 2019; Basso et al., 2019; Beknazarova et al., 2019; Wulcan et al., 2019; Aupalee et al., 2020; Sanpool et al., 2020; Salant et al., 2021; Repetto et al., 2022; Borrás et al., 2023; Nosková et al., 2023, 2024; Beiromvand et al., 2024; de Ree et al., 2024). In contrast, *cox*1 lineage B has been reported exclusively in dogs from Southeast Asia (Jaleta et al., 2017; Nagayasu et al., 2017), South Asia (de Ree et al., 2024) and Australia (Beknazarova et al., 2019). An exception to this is the recent identification of a human isolate from Bangladesh carrying the dog-specific mitochondrial (*cox1* lineage B) and nuclear (HVR-IV haplotype V) genotypes, suggesting possible incidental zoonotic transmission (de Ree et al., 2024).

To date, genotyping surveys have identified 6 *18S rRNA* HVR-IV haplotypes (A, B, C, E, J, V) and 11 HVR-I haplotypes (I–XII, XV) within *S. stercoralis* (Bradbury et al., 2021). *18S rRNA* HVR-IV haplotype A is the predominant genotype detected in humans, dogs and NHPs worldwide (Bradbury et al., 2021). *18S rRNA* HVR-IV haplotype B has been reported in dogs from Cambodia (Jaleta et al., 2017) and Australia (Beknazarova et al., 2019), while haplotype V was identified in canine isolates from Bangladesh and is likewise considered dog-specific (de Ree et al., 2024). Other haplotypes include C, isolated from a human infection in Southeast Asia but maintained via serial passage through laboratory dogs in Australia (Putland et al., 1993); E, detected in canine and human strains from Australia and China, respectively (Beknazarova et al., 2019; Zhou et al., 2019); and J, reported in a human strain from the USA (Barratt et al., 2019). Due to the high variability of *18S rRNA* HVR-I haplotypes within host-specific *S. stercoralis* isolates, this region has not been considered a reliable marker for inferring host specificity (Bradbury et al., 2021).

Two population genomics studies have provided new insights into the host specificity of *S. stercoralis* (de Ree et al., 2024; Liu et al., 2025). de Ree et al. (2024) analysed whole-genome sequences from worms isolated from 7 human hosts and 1 dog in Bangladesh. Their results largely support the previously described 2-lineage population structure of *S. stercoralis*. However, 2 dog-derived isolates possessed a nuclear genome containing *18S rRNA* HVR-IV haplotype V and a mitochondrial genome corresponding to *cox*1 lineage A, suggesting historical inter-lineage hybridization (de Ree et al., 2024). In a second, larger-scale study, Liu et al. (2025) examined *S. stercoralis* from sympatric human (*n* = 26) and dog (*n* = 12) populations in Bangladesh, Cambodia and Thailand. Whole-genome analysis revealed that human- and dog-derived *S. stercoralis* form 2 largely distinct populations that diverged genetically approximately 8000–12 000 years ago. Nevertheless, evidence of introgression was detected, consistent with the findings of de Ree et al. (2024), suggesting that reproductive isolation is incomplete. Also, 1 dog harboured a worm possessing human-type mitochondrial and nuclear genomes, supporting the potential for occasional cross-species transmission (Liu et al., 2025). Together, these studies support the hypothesis first proposed by Nagayasu et al. (2017), that *S. stercoralis* originally parasitized canids and subsequently adapted to humans and other hosts, likely following the domestication of dogs. Although the human- and dog-infecting *S. stercoralis* have diverged genetically, they have not undergone complete speciation.

In a separate study investigating the clinical relevance of *S. stercoralis* genotypes, Repetto et al. (2022) analysed *cox*1 haplotypes in patients from South America and the Caribbean. No significant differences in clinical presentation were observed between haplotypes; however, reactivation of strongyloidiasis following ivermectin treatment was significantly less frequent in infections with *cox*1 haplotypes carrying the I152V mutation. These findings require validation in larger, geographically diverse cohorts.

#### Strongyloides fuelleborni fuelleborni

Genetic characterization of *S. f. fuelleborni* has been conducted on isolates from humans and NHPs across multiple African and Asian countries, as well as from imported African NHPs in St. Kitts and a UK safari park (Table 3). Most studies targeted partial *cox*1 and *18S rRNA*, while 3 examined the complete mitochondrial genome (Ko et al., 2023; de Ree et al., 2024; Richins et al., 2025). Across studies, *S. f. fuelleborni* isolates consistently showed allopatric clustering. At the *cox1* locus, sequences grouped broadly into African and Asian clades, with each geographic cluster (A–I) containing isolates from sympatric human and NHP populations (Hasegawa et al., 2016; Thanchomnang et al., 2017, 2019; Frias et al., 2018; Barratt et al., 2019; Janwan et al., 2020; Ko et al., 2023; de Ree et al., 2024; Richins et al., 2025). Human isolates from PNG grouped within the Asian clade, suggesting possible historical introduction via human migration (Zhao et al., 2025c). At the *18S rRNA* locus, 5 HVR-I haplotypes (XII–XVII) and 11 HVR-IV haplotypes (K–U) have been identified (Barratt et al., 2019; Richins et al., 2023, 2025). Asian-Pacific strains, regardless of host species, consistently belonged to HVR-IV haplotype S and HVR-I haplotype XIV (Sato et al., 2007; Hasegawa et al., 2009; Thanchomnang et al., 2017; Barratt et al., 2019; Janwan et al., 2020; de Ree et al., 2024; Zhao et al., 2025c). In contrast, African strains showed greater haplotypic diversity at both loci (Hasegawa et al., 2009, 2010, 2016; Barratt et al., 2019; Richins et al., 2023, 2025).

Hasegawa et al. (2016) hypothesized that *S. f. fuelleborni* diversified genetically through geographic dispersal and isolation associated with the migration of Old World primates from Africa to Asia by the end of the Miocene. If this hypothesis is correct, it becomes important to assess whether African and Asian *S. f. fuelleborni* differ sufficiently, both genomically and morphologically, to warrant recognition as distinct taxa. Viney et al. (1991) described subtle morphological differences between *S. f. kellyi* from PNG and *S. f. fuelleborni* from Africa. No morphological studies of *S. f. fuelleborni* from Asia have been reported. If *S. f. kellyi* is truly synonymous with the Asian clade of *S. f. fuelleborni*, these morphological distinctions may justify subspecific differentiation between the African and Asian lineages (Zhao et al., 2025c).

To investigate the evolutionary history of *S. f. fuelleborni*, Ko et al. (2023) examined mitochondrial gene arrangement patterns in Asian isolates and identified 2 distinct types. Type A, observed in wild isolates from Myanmar and Japan, contained a single tRNA-Met gene. Type B, found in captive NHPs from Japanese zoos, included 2 copies of this gene. Ko et al. (2023) suggested that Type A may represent the ancestral state of the *S. f. fuelleborni* mitochondrial genome. Subsequently, Type A was also identified in human isolates from Bangladesh (de Ree et al., 2024). Richins et al. (2025) analysed mitochondrial genomes from captive NHPs of putative African origin housed in a UK safari park and identified a novel arrangement, designated Type C. This arrangement also included 2 copies of the tRNA-Met gene but differed from Types A and B in overall gene order. Additionally, the mitochondrial genomes of these UK isolates were substantially larger (∼24 kilobases) than those of Asian *S. fuelleborni* (∼16 kilobases), due to expanded intergenic regions of unknown function. While the findings of Richins et al. (2025) support a distinction between Asian and African clades of *S. f. fuelleborni*, they should be interpreted with caution, as the worms were derived from zoo-kept African NHPs of unknown duration in captivity. Further investigation using wild-caught African isolates is necessary to determine whether the mitochondrial arrangement observed in the UK samples reflects natural variation or adaptations acquired during captivity.

#### Strongyloides fuelleborni kellyi

Prior to the molecular identification of *S. f. fuelleborni* in New Guinea (Zhao et al., 2025c), *S. f. kellyi* was considered the only non-*S. stercoralis Strongyloides* nematode infecting humans in the region (Zhao et al., 2025a). The only sequence data attributed to *S. f. kellyi* at that time was a 330 bp fragment of *18S rRNA* (Dorris et al., 2002), which includes the HVR-I (Hasegawa et al., 2009). This sequence (AJ417029) was found to be identical to those of *S. cebus* (AJ417025) and *S. papillosus* (AJ417027) and clustered separately from *S. f. fuelleborni* and *S. stercoralis* in the same analysis (Dorris et al., 2002). Zhao et al., (2025c) later demonstrated that the genospecies identified by Dorris et al. (2002) was inconsistent with the formal designation of *S. f. kellyi* proposed by Viney et al. (1991) and likely represents an undescribed *Strongyloides* sp. infecting humans in PNG.

#### Strongyloides planiceps

Fourteen sequences of *S. planiceps* are available from three studies (Hasegawa et al., 2009, 2010; Ko et al., 2020), targeting *cox1* (*n* = 9), *18S rRNA* (*n* = 4) or *28S rRNA* (*n* = 1) loci. All sequenced isolates were obtained from raccoon dogs (*Nyctereutes procyonoides*) in Japan. Phylogenetic analyses of *cox*1 sequences (Hasegawa et al., 2010; Ko et al., 2020) and concatenated *18SrRNA* and *28S rRNA* sequences (Ko et al., 2020) confirmed that *S. planiceps* is genetically distinct from *S. stercoralis* and *S. f. fuelleborni*. Intraspecific genetic diversity within *S. planiceps* has not yet been studied.

#### Strongyloides species of unknown identity

Published sequences of unidentified *Strongyloides* spp. from dogs and cats include 5 *cox1* sequences and 2 *18S rRNA* sequences from Australian dogs (Beknazarova et al., 2019) and 32 sequences of *cox1* (*n* = 28), *18S rRNA* (*n* = 2), *28S rRNA* (*n* = 1) and mitochondrial genome (*n* = 1) from cats in Myanmar (Ko et al., 2020).

Beknazarova et al. (2019) conducted metabarcoding of faecal DNA from 20 dogs and 4 humans in remote northern Australia. In 2 dogs, they identified a *Strongyloides* sp. that was phylogenetically basal to all known *S. stercoralis* isolates at the *cox*1 locus. One of these dogs also harboured unique *18S rRNA* HVR-I and HVR-IV haplotypes (genotype VIII/F). These findings should be interpreted with caution, as they may represent transient passage of *Strongyloides* DNA from other hosts consumed in the dog’s diet or from environmental sources rather than true infections. Nonetheless, the possibility of a novel, undescribed species, or a genetically distinct *S. stercoralis* lineage endemic to Australian dogs, cannot be ruled out.

Ko et al. (2020) sequenced partial *cox1, 18S rRNA, 28S rRNA* and the mitochondrial genome from 70 *Strongyloides* isolates obtained from 19 cats in Myanmar. All *18S rRNA* sequences were identical. Phylogenetic analysis of mitochondrial protein-coding genes and *cox1* placed these isolates in a sister clade to *S. stercoralis*. Ko et al. (2020) suggested that this cat-derived *Strongyloides* sp. may represent *S. felis*, but the absence of morphological characterization makes this conclusion speculative. Similarly, Jitsamai (2019) analysed a 708 bp fragment of *18S rRNA* from *Strongyloides* isolates obtained from cats in Thailand and found that they formed a sister group to *S. stercoralis* and *S. procyonis*. Although morphological examination of the free-living stages supported identification as *S. felis*, this diagnosis is dubious due to deviations from established morphological criteria. Moreover, the *18S rRNA* sequences from this study are not publicly available in GenBank, which precludes further verification.

### Limitations of sequence-based taxonomy

A fundamental limitation in the molecular taxonomy of *Strongyloides* is that species have historically been defined on the basis of morphological characters of the adult stages. Most contemporary sequence data, however, are generated from DNA extracted from faeces of infected hosts or from larvae isolated from faeces (Table 3), stages that do not permit reliable speciation based on morphology (Little, 1966a; Speare, 1986, 1989). As a result, sequences derived from these sources cannot be confidently assigned to morphologically defined taxa, except in the rare cases where the corresponding adult worms have been recovered and morphologically identified. Despite this, such sequences are frequently assigned species names, typically based on the host species from which the sample was collected. This introduces a circularity: host identity is used to infer species identity of the sequence, which then becomes the basis for conclusions about host specificity, population structure or species boundaries (Liu et al., 2025). Molecular data should therefore be interpreted with caution. Ideally, sequence-based assignments should be validated through morphological identification of the corresponding adult parasitic or free-living stages. Where such verification is not possible, molecular identifications should be regarded as provisional, and any taxonomic or phylogenetic analyses should explicitly acknowledge this limitation.

A further limitation arises from the reliance on single mitochondrial or nuclear loci for phylogenetic inference in much of the existing literature (Table 3). These loci represent only a minute fraction of the genome and often contain few phylogenetically informative sites, sometimes as few as 20–30 bp among several hundred analysed, as observed for *18S rRNA* HVRs (Hasegawa et al., 2009). While these regions are highly conserved and generally effective for distinguishing major lineages and inferring interspecific relationships (Hasegawa et al., 2009, 2010; Barratt et al., 2019; Bradbury et al., 2021), they may not reliably resolve intraspecific diversity or fine-scale population structure (Liu et al., 2025). In contrast, whole-genome analysis examines thousands to millions of informative sites across the genome, providing substantially greater resolving power and enabling more robust, statistically supported phylogenetic and population genetic inferences (Al-Jawabreh et al., 2024b). Nevertheless, its use remains limited, largely due to technical complexity, cost and resource constraints.

The complex reproductive biology of *Strongyloides* spp. poses additional challenges for molecular taxonomy. Parasitic females reproduce obligately by mitotic parthenogenesis, whereas facultative free-living adults reproduce sexually (Viney, 2006; Streit, 2008). Development can follow an asexual (direct) or sexual (indirect) route, influenced by environmental conditions and host factors, and this balance may shift over evolutionary time (Harvey et al., 2000). Principally asexual populations of *S. stercoralis* have been reported from Asia (Kikuchi et al., 2016; Zhou et al., 2019). Because parthenogenesis is obligatory in the parasitic stage, patterns of genetic divergence may deviate from expectations under species concepts that assume regular sexual reproduction and gene flow (Viney, 2006; Streit, 2017). For example, population genomic analysis of *S. ratti* from the UK revealed a swarm of highly divergent clonal genotypes that are nonetheless regarded as a single species (Cole et al., 2023). This pattern illustrates how asexual reproduction can facilitate the long-term persistence of genetically distinct lineages within a nominal taxon. This, in turn, creates a taxonomic dilemma: although these lineages are genetically well differentiated, classical species concepts based on reproductive isolation offer little guidance where sexual reproduction is rare or absent (Streit, 2017). These issues have important implications for interpreting population structure and host specificity in *Strongyloides* spp. when applying population genetic or population genomic approaches (Cole et al., 2023; Liu et al., 2025). Whether alternative species concepts can be meaningfully applied to *Strongyloides* remains an open question.

## Conclusion

The taxonomy of *Strongyloides* in humans and companion animals has historically relied on morphology but is increasingly informed by molecular genetics. Integrating morphological and genomic data offers the greatest potential for resolving taxonomic ambiguities within the genus. Future research should prioritize genomic characterization of diverse *Strongyloides* strains from humans and animals, refine reference genomes, optimize DNA barcoding protocols and undertake detailed comparative morphological analyses to support taxonomic delineation. Additionally, efforts must be made to preserve and sustain morphological expertise to ensure its continued relevance alongside advancing molecular techniques.

## Figure 1 Long description

The image shows five different intestinal nematode eggs, each labeled with its name and size indicated in micrometres on the y-axis. From left to right, the eggs are Strongyloides fuelleborni, Hookworm, Oesophagostomum spp., Ternidens deminutus and Trichostrongylus spp. The y-axis ranges from 0 to 100 micrometres, providing a scale for comparison of egg sizes. Each egg has distinct visual characteristics, with variations in texture and pattern visible within the shells.

Navigate back to Figure 1.

## Figure 2 Long description

The image shows three sections. The first section (a) illustrates various stomal shapes: round, dumbbell, dumbbell with oesophageal teeth, X-shaped, hexagonal and rectangular. The second section (b) depicts ovary types: straight and spiral. The third section (c) shows tail morphologies: bluntly rounded, narrowly tapered and narrowly tapered to a point.

Navigate back to Figure 2.

## Figure 3 Long description

The image shows four panels labeled a, b, c and d, each depicting the full body of a parasitic female nematode. Insets highlight the stoma and tail regions. In panel a, the stoma is shown in en face, lateral and dorsoventral views. Panel b displays similar views with a different stoma shape. Panel c presents another stoma configuration in the same views. Panel d shows yet another stoma type, again in en face, lateral and dorsoventral views. Each nematode illustration includes a scale bar indicating 100 micrometers for the full body and 10 micrometers for the stoma views.

Navigate back to Figure 3.

## Figure 4 Long description

The image shows four illustrations labeled a, b, c and d. Illustration a depicts the absence of post-vulval body narrowing. Illustration b shows the presence of post-vulval body narrowing. Illustration c illustrates the absence of posterior vulval rotation, with the vulval slit forming an angle of 90 to 100 degrees with the longitudinal body axis. Illustration d shows the presence of posterior vulval rotation, with the vulval slit forming an angle greater than 100 degrees with the longitudinal body axis.

Navigate back to Figure 4.

## Figure 5 Long description

The image A shows three illustrations of spicule morphology labeled as straight, convex and concave. The image B shows two diagrams of tail structures with numbered caudal papillae: (1) subventral preanal papilla, (2) anterior adanal papilla, (3) posterior adanal papilla, (4) lateral papilla, (5) subventral postanal papilla and (6) subdorsal postanal papilla. The left diagram shows alignment of the subventral preanal papilla with the adanal papillae, while the right diagram shows a different arrangement, indicated by a dashed line and an arrow pointing to the preanal organ.

Navigate back to Figure 5.

## Table 1 Long description

The table compares morphometric data of various Strongyloides species infecting humans, dogs, and cats, focusing on egg size, larval stages, and adult forms. S. tumefaciens exhibits the largest parasitic female body length at 5000 micrometers, whereas S. felis has the smallest rhabditiform larvae body length ranging from 217 to 238 micrometers. Egg sizes vary significantly, with S. tumefaciens having the largest eggs at 114 to 124 micrometers by 62 to 68 micrometers. Notably, data for some species and measurements are missing, such as the rhabditiform larvae body width for S. felis. These variations highlight the diversity in size and morphology among the species, which may influence their biological and ecological roles.

Navigate back to Table 1.

## Table 2 Long description

The table compares morphological features of Strongyloides species infecting humans, dogs, and cats, focusing on stages in fresh feces, stomal shape, circumoral lobes, ovary type, tail shape, and other characteristics. S. stercoralis and S. felis exhibit rhabditiform larvae, while others show larvated eggs. Stomal shapes vary from hexagonal in S. stercoralis to dumbbell-shaped in S. planiceps. Circumoral lobes range from 0 in S. f. fuelleborni to 6 in S. stercoralis and S. felis. Ovary types are either straight or spiral, with S. planiceps and S. f. fuelleborni showing spiral ovaries. Tail shapes are predominantly narrowly tapered or bluntly rounded. Notably, S. stercoralis and S. felis have autoinfective larvae, while S. tumefaciens parasitic females are found in the large intestine. These morphological differences are crucial for species identification and understanding their parasitic behavior.

Navigate back to Table 2.

## Table 3 Long description

This table provides a comprehensive overview of phylogenetic and population genetic studies on Strongyloides species infecting humans, dogs, and cats. It includes data from multiple countries, highlighting the host species, DNA sources, gene regions analyzed, and sequencing methods used. Key findings include the use of Sanger and Illumina sequencing methods to analyze gene regions such as 18S rRNA, cox1, and ITS-1. The studies cover a wide range of geographical locations, including the USA, Iran, Japan, and Australia, among others. Notably, the table reveals a focus on the infective third-stage larvae and free-living adults as DNA sources. The data also indicate a trend towards using whole genome sequencing in more recent studies. Some entries lack specific data, such as GenBank accessions, which may limit the interpretation of certain studies.

Navigate back to Table 3.
